# Impulsive Release Strategies for Wolbachia-Infected Mosquitoes Under Temperature-Induced Infection Loss

**DOI:** 10.1007/s11538-026-01761-w

**Published:** 2026-09-28

**Authors:** Jéssica C. S. Alves, Christian E. Schaerer, Cláudia P. Ferreira

**Affiliations:** 1 Institute of Mathematics and Statistics, University of São Paulo, São Paulo, São Paulo 05508-090 Brazil; 2 Polytechnic School, National University of Asunción, P.O. Box 2111 SL, San Lorenzo, Central Paraguay; 3 Institute of Biosciences, São Paulo State University, Botucatu, São Paulo 18618-689 Brazil

**Keywords:** Impulsive system, Release amount, Loss of infection, High temperature, Population dynamics

## Abstract

The release of *Wolbachia*-infected mosquitoes is a promising strategy for controlling *Aedes aegypti* populations, although high-temperature exposure may temporarily reduce infection levels and compromise long-term persistence. We propose a population-dynamics model based on impulsive differential equations that incorporates cytoplasmic incompatibility, periodic mosquito releases, and temperature-driven infection loss. Analytical results establish fundamental qualitative properties of the continuous and impulsive models, derive conditions for the existence and stability of periodic solutions, and provide an explicit release threshold for the successful establishment and long-term persistence of the infected population. Numerical simulations validate the theoretical results and compare the performance of the wMelPop, wMel, and wAlbB strains under different thermal scenarios, highlighting the influence of strain-specific fitness costs and thermal tolerance. Additional simulations investigate temperature-dependent demographic variation and imperfect maternal transmission, extending the analysis beyond the assumptions of the analytical framework. The proposed approach provides a tractable mathematical framework for analyzing and designing impulsive *Wolbachia* release strategies under environmentally driven infection loss.

## Introduction

Mosquito population control is a common strategy for reducing the transmission of vector-borne diseases. In this work, we specifically consider the dynamics of *Aedes aegypti*, the vector of several arboviruses, including dengue, Zika, chikungunya, and yellow fever (Ahebwa et al. 2023; Zardini et al. 2024). This species is now present in most tropical and subtropical regions and has the potential to establish itself in more than 80% of countries across the globe (Cheong et al. 2025; Lim et al. 2025). Current control methods include the use of insecticides, mechanical elimination of breeding sites, and biological control approaches (Dorigatti et al. 2018; Lees et al. 2023; Onen et al. 2023).

Biological control comprises several techniques, including the use of natural predators, parasitoids, and specific pathogens that affect mosquito development and survival. Among the most common approaches is the introduction of natural predators, such as certain fish species, including *Gambusia affinis*, which consume mosquito larvae in aquatic environments (Steven et al. 2021). Another strategy involves predatory mosquitoes and parasitoid insects: larvae of mosquitoes of the genus *Toxorhynchites* prey on other mosquito larvae (Sukupayo et al. 2024), while parasitoid wasps, such as *Trichogramma*, oviposit on mosquito eggs, contributing to population suppression (Parra and Coelho 2022). Additionally, insecticides remain an important component of mosquito control. However, large-scale application raises environmental concerns, including the evolution of insecticide resistance, which has been reported in several countries and compromises the effectiveness of mosquito-borne disease control efforts (Carson et al. 2023).

In addition to these biological and chemical approaches, genetic control techniques, such as the Sterile Insect Technique (SIT), have been widely applied in the management of agricultural pests (Zheng et al. 2019) and adapted to mosquito sterilization (Almeida et al. 2022; Bliman et al. 2019; Huang et al. 2017, 2021; Li and Ai 2020). Finally, an emerging biological control strategy involves the release of mosquitoes carrying *Wolbachia*, a symbiotic bacterium that reduces mosquito vector competence and limits arbovirus transmission without requiring genetic modification of the mosquito population (Dorigatti et al. 2018; Ross 2021; Pinto et al. 2021; Dos Santos et al. 2022; Zara et al. 2016; Gesto et al. 2021).

The interaction between wild mosquitoes and those infected with *Wolbachia* or genetically modified mosquitoes has been extensively investigated in the literature (Almeida et al. 2022; Bliman et al. 2019; Gonzales et al. 2025; Orozco-Gonzales et al. 2024; Almeida et al. 2019a; Cardona-Salgado et al. 2020; Lopes et al. 2023; Yu and Zheng 2019). Most of this literature relies on mathematical modeling to analyze population dynamics, employing systems of ordinary differential equations (Almeida et al. 2022, 2019a; Campo-Duarte et al. 2017; Benedito et al. 2023), delay differential equations (Lopes et al. 2023; Santos Benedito et al. 2020; Li and Ai 2020), and discrete-time formulations (Blackwood et al. 2018; Ufuktepe 2022; Yu and Zheng 2019), among others. In addition, optimal control frameworks have been widely adopted to design efficient intervention strategies (Almeida et al. 2022; Gonzales et al. 2025; Campo-Duarte et al. 2017, 2018; Almeida et al. 2019b). These modeling approaches aim to reduce the wild mosquito population and, consequently, mitigate disease transmission.

While much of the existing work focuses on continuous release strategies of *Wolbachia*-infected mosquitoes (Cardona-Salgado et al. 2020; Lopes et al. 2023; Benedito et al. 2023; Ogunlade et al. 2020), alternative formulations based on impulsive releases have also been proposed (Gonzales et al. 2025; Almeida et al. 2024; Li et al. 2024; Liu et al. 2023; Dianavinnarasi et al. 2021). Periodic and impulsive release strategies appear more frequently in the context of sterile insect techniques (Huang et al. 2021; Li and Ai 2020) and in applications to other insect populations and pest control problems (Liu et al. 2023; Alves et al. 2026; Pei et al. 2018; Wang et al. 2011). These approaches are designed to optimize the timing and magnitude of releases, enhancing control efficiency and improving the feasibility of real-world interventions. Field deployment programs have also involved additional or supplementary releases to support the establishment and persistence of *Wolbachia*-infected mosquito populations (Ryan et al. 2020; Nazni et al. 2019; Velez et al. 2023).

This work specifically addresses the control of *Aedes aegypti*, with a particular focus on utilizing the endosymbiotic bacterium *Wolbachia* as a biological control strategy. Several experimental and field studies have shown that the release of *Wolbachia*-infected mosquitoes can substantially reduce vector competence and, consequently, the transmission of arboviruses (Ross 2021; Dos Santos et al. 2022; Zara et al. 2016). These findings support the use of Wolbachia as an effective tool for the long-term control of Aedes aegypti populations (Pavan et al. 2025).

In this article, we model the interaction between wild *Aedes aegypti* mosquitoes and mosquitoes carrying the *Wolbachia* bacterium, incorporating the mechanism of cytoplasmic incompatibility, which reduces the reproductive success of uninfected females when mating with infected males (Kaur et al. 2024). The model also includes two impulsive events: one representing the loss of *Wolbachia* infection due to high-temperature exposure, and the other corresponding to the periodic release of *Wolbachia*-infected mosquitoes. Within this framework of impulsive differential equations, we analyze the system dynamics to determine conditions that ensure the fixation of *Wolbachia*-carrying mosquitoes in the population.

Field and laboratory evidence indicate that high temperatures can temporarily reduce *Wolbachia* density and weaken cytoplasmic incompatibility in *Aedes aegypti*, particularly in environments subject to intense thermal fluctuations (Ross et al. 2017; Gunasekaran et al. 2022). For the wMel strain, heatwaves have been associated with decreases in infection frequencies, with values falling to 83% in larvae sampled directly from field habitats and to 88% in eggs collected from ovitraps, before recovering toward baseline levels in subsequent months (Ross et al. 2020). Experimental studies also report drastic reductions in *Wolbachia* density when immature stages experience elevated temperatures, with levels dropping to below 0.1% of controls after seven days of heat exposure beginning at the egg stage, followed by partial recovery once temperatures return to milder ranges (Gunasekaran et al. 2022; Ulrich et al. 2016). These observations motivate the inclusion in our model of a redistributive impulsive event representing episodic loss of infection due to high-temperature exposure, capturing reductions in the proportion of infected mosquitoes, transferring this proportion to the wild mosquito population, and allowing for their gradual reestablishment over time.

Temperature effects, however, are strain-dependent, with direct implications for the magnitude and frequency of the impulsive loss term in the proposed model. The wMel strain, while capable of blocking dengue and Zika transmission, shows sensitivity to elevated temperatures and exhibits reduced stability under heat stress (Ross et al. 2017; Ulrich et al. 2016; Ant et al. 2018). The wMelPop strain is even more susceptible, displaying pronounced fitness costs and sharp declines in density when exposed to high temperatures (Ross et al. 2019, 2017), suggesting a larger and more frequent infection-loss impulse. In contrast, the wAlbB strain demonstrates greater tolerance to thermal stress, maintaining comparatively higher densities under fluctuating or elevated temperatures (Gunasekaran et al. 2022; Mancini et al. 2021), implying a weaker impulsive reduction in infection levels. Since both pathogen-blocking capacity and thermal resilience influence persistence and replacement dynamics, we summarize these characteristics in Table 1 to support the interpretation of the model parameters and their biological relevance.

**Table 1:** Key biological traits of *Wolbachia* strains: virus blocking, heat tolerance, and fitness effects

Strain	Virus block	Heat tolerance	Impact on host fitness	References
wMelPop	Very high	Low	Strong reduction in fecundity, egg viability, and longevity	(Ross et al. 2019, 2017; Ritchie et al. 2015)
wMel	Moderate	Low	Moderate reduction in fecundity and egg viability; little effect on longevity	(Ulrich et al. 2016; Mancini et al. 2021; Maciel-de-Freitas et al. 2024)
wAlbB	High	Moderate/High	Mild effects; fertility may drop under heat stress	(Gunasekaran et al. 2022; Ant et al. 2018; Axford et al. 2016)

To address this problem, we formulate a population dynamics model governed by impulsive differential equations that incorporates two distinct impulsive mechanisms: the periodic release of *Wolbachia*-infected mosquitoes and the temperature-driven loss of infection. This modeling approach extends existing impulsive frameworks by coupling operational interventions with environmentally induced perturbations. We establish analytical conditions for the existence, characterization, and stability of periodic solutions, and derive explicit threshold conditions that link the magnitude and timing of release events to the intensity and frequency of thermal infection loss. These results provide theoretical criteria guaranteeing long-term persistence of *Wolbachia* infection in the mosquito population.

In addition to the theoretical analysis, we perform numerical simulations to validate the derived threshold conditions and to investigate how episodic high-temperature events influence infection dynamics. The simulations illustrate the interplay between release schedules, environmental stress, and strain-specific biological traits, while also examining two biologically relevant extensions that are not included in the analytical framework, namely temperature-dependent demographic variation and imperfect maternal transmission. These additional experiments provide further insight into the sensitivity of the proposed release strategy to assumptions commonly adopted in mathematical models of *Wolbachia* population replacement.

The main contributions of this work are the analytical characterization of the impulsive model and the derivation of an explicit threshold for the number of *Wolbachia*-infected mosquitoes that must be released at each intervention time τ. This threshold guarantees the successful establishment of the infected mosquito population and the elimination of the wild population in the absence of temperature-induced infection loss. Furthermore, when episodic infection loss due to high temperatures is incorporated into the model, the obtained condition ensures that the wild mosquito population can be maintained arbitrarily close to zero, provided that the release magnitude satisfies the derived threshold. Complementing the analytical results, additional numerical simulations investigate temperature-dependent demographic variation and imperfect maternal transmission, demonstrating how these biologically relevant extensions influence infection persistence and the effectiveness of the proposed release strategy.

The remainder of this paper is organized as follows. Section 2 presents the interaction model between wild and *Wolbachia*-infected *Aedes aegypti* mosquitoes, establishing the fundamental properties of the solutions, including existence, uniqueness, positivity, boundedness, and the local stability of equilibrium states. Section 3 introduces the impulsive population-dynamic model and provides the theoretical analysis of the coupled impulsive system, where both release interventions and temperature-induced infection loss are formally described. In this section, we derive threshold conditions ensuring long-term infection persistence. Section 4 presents numerical simulations supporting the analytical results, compares the performance of different *Wolbachia* strains under distinct temperature-induced infection-loss scenarios, and investigates the effects of temperature-dependent demographic variation and imperfect maternal transmission. Finally, Sect. 5 summarizes the main conclusions and discusses the applicability and limitations of the proposed framework.

## Model Formulation

We propose the following mathematical model to characterize the population dynamics of *Aedes aegypti* mosquitoes, distinguishing between the wild population and the one infected with *Wolbachia*:

1 $$\begin{aligned} {\left\{ \begin{array}{ll} \dfrac{dS_1}{dt} = S_1\psi _1\left( 1 - \dfrac{S_1 + S_2}{K} \right) \left( \dfrac{S_1+(1-\gamma )S_2}{S_1+S_2} \right) - \delta _1 S_1, \\ \dfrac{dS_2}{dt} = S_2\psi _2 \left( 1 - \dfrac{S_1 + S_2}{K} \right) - \delta _2 S_2. \end{array}\right. } \end{aligned}$$

with non-negative initial conditions and positive parameters, $S_1(t)$ represents the population of wild *Aedes aegypti* mosquitoes, while $S_2(t)$ represents the population of *Wolbachia*-infected mosquitoes over time *t*. The parameters $\psi _i$ and $\delta _i$ denote the birth and death rates of $S_1$ and $S_2$, respectively, for i=1,2, whereas $\gamma \in [0,1]$ is the cytoplasmic incompatibility parameter. The parameter *K* corresponds to the shared environmental carrying capacity of wild and infected populations.

The model in (1) was constructed to capture the main biological mechanisms governing the interaction between wild and *Wolbachia*-infected *Aedes aegypti* populations. The logistic-type terms reflect the limitation imposed by resource competition, assuming that both populations share the same environmental carrying capacity *K*. This shared constraint expresses the ecological competition for breeding sites and food resources, which are finite within the habitat.

The multiplicative structure of the growth terms ensures that population change is proportional to the current population size, following standard formulations in population dynamics. The additional factor

$$ \left( \frac{S_1+(1-\gamma )S_2}{S_1+S_2}\right) $$

in the equation for $S_1$ represents the cytoplasmic incompatibility (CI) effect: when *Wolbachia*-infected males mate with wild females, a fraction γ of the eggs fail to develop (Kaur et al. 2024). Thus, (1-γ) quantifies the viable offspring fraction produced in mixed matings, reducing the reproductive success of the wild population.

The infected population, on the other hand, does not experience this reduction, as *Wolbachia* transmission is assumed to be maternal and, in this case, fully efficient. Therefore, the growth of $S_2$ is described by a simpler logistic-like term, driven by $\psi _2$ and limited by the same carrying capacity *K*. Maternal transmission of *Wolbachia* is assumed to be perfect, and therefore, vertical transmission failure is not considered. Possible mechanisms leading to infection loss are introduced and discussed in the following section.

The assumption of perfect maternal transmission is adopted here as an idealized baseline, commonly used in models of *Wolbachia* dynamics under stable environmental conditions. However, experimental evidence indicates that elevated temperatures may reduce *Wolbachia* density and compromise vertical transmission, leading to the production of uninfected offspring by infected females.

In the present model, these effects are not explicitly included in the continuous dynamics, being represented in an aggregate way through the impulsive infection-loss term. A limitation of this modeling approach is that it does not capture the generational effects associated with imperfect maternal inheritance, which may lead to optimistic predictions of persistence under strong thermal stress. To evaluate the impact of this assumption, simulations incorporating a transmission efficiency parameter $\xi \in (0,1]$ are presented in Sect. 4.

The model is not stage-structured and therefore does not explicitly describe the effects of thermal stress on eggs, larvae, or pupae. Experimental evidence (Ross et al. 2017; Gunasekaran et al. 2022) indicates that these stages may be particularly sensitive to high temperatures, which can affect Wolbachia density and the infection frequency observed in the adult population.

In this context, the impulsive redistribution term should be interpreted as an aggregate representation of temperature-induced infection loss over the development process. As a result, the model cannot distinguish how thermal stress acting on different developmental stages contributes to the observed reduction in infection frequency. Addressing this question would require a stage-structured model, which is beyond the scope of the present work.

For this model, we consider the following assumptions:

2 $$\begin{aligned} \psi _1> \delta _1 \quad \text {and} \quad \psi _2 > \delta _2, \end{aligned}$$

which guarantees that birth rates exceed death rates, allowing population persistence in the absence of competition. Additionally, we assume

3 $$\begin{aligned} \psi _2 < \psi _1, \quad \text {and} \quad \delta _2 > \delta _1, \end{aligned}$$

implying that wild mosquitoes have greater fitness than *Wolbachia*-infected mosquitoes. This assumption is biologically realistic, as infection typically imposes a fitness cost on mosquitoes, reducing reproductive rates and increasing mortality. However, depending on the *Wolbachia* strain, these effects may vary significantly: some strains cause substantial reductions in mosquito fitness, while others exhibit milder impacts or even confer slight advantages under specific environmental conditions.

The interaction between ecological competition and cytoplasmic incompatibility determines whether the infection can coexist with or replace the wild population. The infection’s potential to replace the wild population depends on the interplay between the fitness cost and the reproductive advantage conferred by CI. Under suitable conditions, the system in (1) can exhibit coexistence or complete replacement of the wild population by the infected one, which will be explored in the subsequent analysis.

### Theorem 1

(Existence, Uniqueness, Positivity, and Global Boundedness) Consider the system (1), with non-negative initial conditions, and assume that all parameters $\psi _i$, $\delta _i$, *K*, and γ are positive constants. Then, the following statements hold:

I. There exists a unique local solution $(S_1(t), S_2(t))$ to system (1) for any given nonnegative initial condition $(S_1(0), S_2(0))$.
II. The positive orthant $\mathbb {R}^2_+ = \{(S_1,S_2) \,|\, S_1 \ge 0,\, S_2 \ge 0\}$ is invariant under the flow of (1). In particular, if $S_1(0), S_2(0) \ge 0$, then $S_1(t), S_2(t) \ge 0$ for all t≥0.
III. The solutions of (1) are uniformly bounded for all t≥0.
IV. Consequently, for any nonnegative initial condition, there exists a unique, positive, and bounded solution $(S_1(t), S_2(t))$ of system (1) defined for all t≥0.

The proof of Theorem 1 is given in the Appendix A.

Having established the existence, uniqueness, positivity, and boundedness of solutions, we now investigate the equilibrium points and their stability properties. This analysis characterizes the long-term behavior of the interacting populations and identifies the conditions leading to coexistence or complete population replacement.

### Theorem 2

(Equilibrium Points and Stability) Consider system (1) with non-negative initial conditions. Under assumptions (2) and (3), define

$$ R_1 = \frac{\psi _1}{\delta _1} \quad \text {and} \quad R_2 = \frac{\psi _2}{\delta _2}. $$

The system may exhibit up to three equilibrium points in the region $\mathbb {R}^2_+ \setminus \{(0,0)\}$, whose existence and local stability properties are described below:

- ***Wolbachia***-**free equilibrium** $E_1$:
  $$\begin{aligned} E_1 = \left( K\left( 1 - \frac{1}{R_1}\right) ,\, 0\right) , \end{aligned}$$
  which exists if $R_1 > 1$ and is locally asymptotically stable if
  $$\begin{aligned} R_1 > R_2. \end{aligned}$$
- **Wild-free equilibrium** $E_2$:
  $$\begin{aligned} E_2 = \left( 0,\, K\left( 1 - \frac{1}{R_2}\right) \right) , \end{aligned}$$
  which exists if $R_2 > 1$ and is locally asymptotically stable if
  $$\begin{aligned} R_2 > (1-\gamma )R_1. \end{aligned}$$
- **Coexistence equilibrium** $$E_3$$:
  $$\begin{aligned} E_3 = (\hat{S}_1,\, \hat{S}_2), \end{aligned}$$
  where
  $$\begin{aligned} \hat{S}_1&= \left( 1 - \frac{1}{R_2} \right) \frac{K}{\gamma } \left( \frac{R_2}{R_1} - (1 - \gamma ) \right) \\ \hat{S}_2&= \left( 1 - \frac{1}{R_2} \right) K - \hat{S}_1. \end{aligned}$$
  The equilibrium $E_3$ exists if and only if
  $$\begin{aligned} 1 - \gamma< \frac{R_2}{R_1} < 1, \end{aligned}$$
  and, whenever it exists, it is a saddle point (thus unstable).

The proof of Theorem 2 is given in the Appendix B.

The conditions derived above admit a clear biological interpretation. The threshold $R_1>1$ indicates that a wild mosquito must produce, on average, more than one wild mosquito per generation for the wild population to persist. Similarly, the condition $R_2>1$ establishes the minimum requirement for the persistence of the Wolbachia-infected mosquito population. The inequality $R_1>R_2$ reflects a higher fitness of the wild population compared to the infected population in the absence of cytoplasmic incompatibility. Finally, the term $(1-\gamma )R_1$ represents the reduction in the effective fitness of the wild population induced by cytoplasmic incompatibility.

**Fig. 1:**
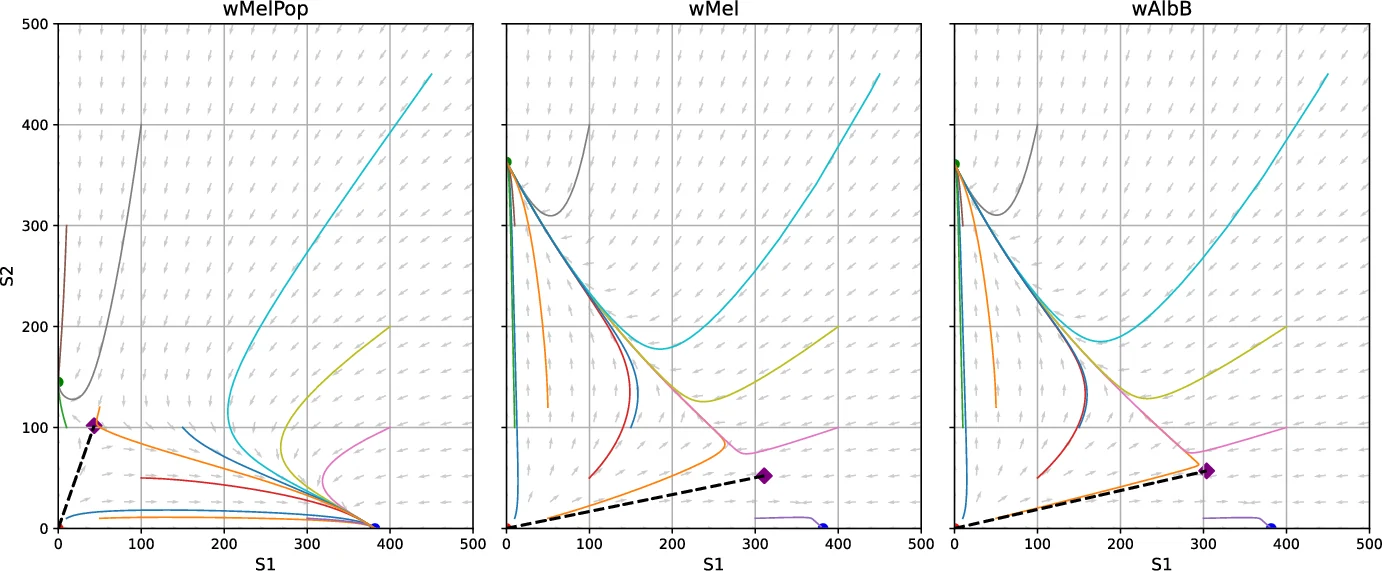
Phase portraits of system (1) for γ=0.95, illustrating the qualitative behavior of trajectories for the three *Wolbachia* strains. Different colors represent trajectories starting from distinct initial conditions. The equilibria $E_1$ (blue), $E_2$ (green), and $E_3$ (purple) are indicated. Parameter values are reported in Tables 3 and 4 (Color figure online)

In Fig. 1, we present the phase portraits of model (1) for different initial conditions, illustrating the trajectories of $S_1$ and $S_2$ relative to the equilibrium points. The results show that the basin of attraction associated with equilibrium $E_2$, which corresponds to the elimination of wild mosquitoes and the persistence of *Wolbachia*-infected individuals, varies according to the parameter set of each strain. In particular, the attraction region for the wMelPop strain is significantly smaller than that observed for the other two strains. This behavior is consistent with the biological characteristics of wMelPop, which is known to reduce key mosquito fitness traits, as reported in Table 1.

The results established in this section characterize the qualitative behavior of the continuous model, including the existence, boundedness, and stability of its equilibrium. Together with the phase portraits, they provide the analytical foundation for the impulsive model introduced in the next section.

## Impulsive Model

Given our objective of studying and formulating release strategies for *Wolbachia*-infected mosquitoes in a specific region, we adopt the framework of impulsive differential equations due to its practical relevance in modeling discontinuous interventions. In this model, we consider two types of periodic impulses: (i) the seasonal loss of infection caused by temperature variations and (ii) the impulsive release of infected mosquitoes.

We consider a fixed release interval τ>0, corresponding to the time between successive mosquito release events. To represent the effects of seasonal temperature variation, we assume that *Wolbachia*-infected mosquitoes may lose the infection when exposed to elevated temperatures. This process is modeled through a redistributive impulse occurring at times t=(n+l)τ, where l∈[0,1] specifies the relative timing of the seasonal infection-loss event within each release cycle. The index $n\in \mathbb {Z}_+$ denotes the release cycle. When l=0 or l=1, the infection-loss event coincides with a mosquito release. At these instants, a proportion $\varphi (t)\in [0,1]$ of the infected population is transferred to the wild population:

$$\begin{aligned} {\left\{ \begin{array}{ll} S_1(t^+) = S_1(t) + \varphi (t) S_2(t), \\ S_2(t^+) = (1 - \varphi (t)) S_2(t), \end{array}\right. } \quad t = (n + l)\tau . \end{aligned}$$

The loss rate φ(t) is modeled as a smooth periodic function representing the seasonal variation in infection loss driven by temperature fluctuations:

4 $$\begin{aligned} \varphi (t) = \varphi _{\max } \left[ 1 + \cos \left( \frac{2\pi (t - t_{\text {peak}})}{365}\right) \right] ^2, \end{aligned}$$

where $\varphi _{\max }$ is a scaling parameter controlling the intensity of temperature-induced infection loss, 365 is the length of the seasonal cycle (one year), and $t_{\textrm{peak}}$ represents the time of the year at which infection loss reaches its maximum. This formulation yields a smooth periodic function satisfying $\varphi (t)\in [0,4\varphi _{\max }]$. To ensure biological consistency, we assume $\varphi _{\max }\in (0,\frac{1}{4}]$, which guarantees that $\varphi (t)\in [0,1]$ for all *t*.

**Fig. 2:**
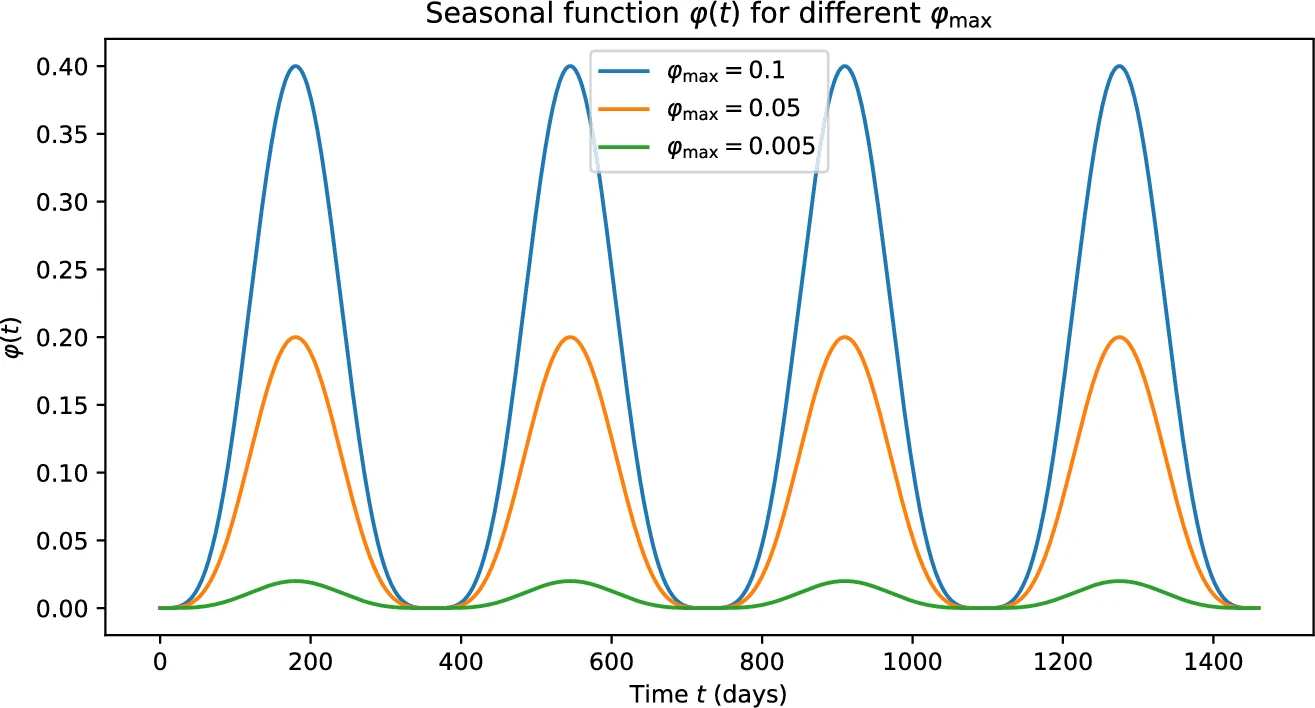
Seasonal function φ(t) defined in (4) plotted for three different values of $\varphi _{\max }$

The impulsive representation is adopted as a mathematical approximation of the temperature-induced infection loss process. Although temperature varies continuously throughout the year, the model aggregates its effect into infection-loss events occurring at specified times within the release cycles. Thus, the impulsive event does not imply that *Wolbachia* loss occurs instantaneously in natural mosquito populations; rather, it represents the cumulative effect of temperature-induced infection loss over the corresponding period. Since the present study does not use real temperature data, the function φ(t) is not intended to reproduce the exact temperature-dependent infection-loss dynamics observed in natural populations. Instead, it provides a tractable framework for investigating how seasonal infection loss may interact with periodic releases of infected mosquitoes (Fig. 2).

At each time instant t=(n+1)τ, a quantity $u_n$ of *Wolbachia*-infected mosquitoes is released into the system. The release acts only on the infected population $S_2$, while the wild population $S_1$ remains unchanged at the moment of intervention:

$$\begin{aligned} {\left\{ \begin{array}{ll} S_1(t^+) = S_1(t), \\ S_2(t^+) = S_2(t) + u_n, \end{array}\right. } \quad t = (n + 1)\tau . \end{aligned}$$

The complete model describing the interaction between wild and *Wolbachia*-infected mosquito populations, incorporating temperature-induced infection loss and impulsive releases of infected mosquitoes, provides a framework for investigating how the release size $u_n$, the release interval τ, and the infection-loss intensity $\varphi _{\max }$ affect the long-term dynamics. The model is given by the following system of impulsive differential equations:

5a $$\begin{aligned}&\left. {\left\{ \begin{array}{ll} \dfrac{dS_1}{dt} = S_1\psi _1\left( 1 - \dfrac{S_1 + S_2}{K} \right) \left( \dfrac{S_1+(1-\gamma )S_2}{S_1+S_2}\right) - \delta _1 S_1, \\ \dfrac{dS_2}{dt} = S_2\psi _2 \left( 1 - \dfrac{S_1 + S_2}{K} \right) - \delta _2 S_2, \end{array}\right. } \right. \hspace{-1cm}\text {if } t \ne (n + l)\tau , \text  t \ne (n + 1)\tau . \end{aligned}$$

5b $$\begin{aligned}&\left. {\left\{ \begin{array}{ll} S_1(t^+) = S_1(t) + \varphi (t) S_2(t), \\ S_2(t^+) = (1 - \varphi (t)) S_2(t), \end{array}\right. } \right. \text {if } t = (n + l)\tau . \end{aligned}$$

5c $$\begin{aligned}&\left. {\left\{ \begin{array}{ll} S_1(t^+) = S_1(t), \\ S_2(t^+) = S_2(t) + u_n, \end{array}\right. } \right. \text {if } t = (n + 1)\tau . \end{aligned}$$

Within the impulsive differential equations framework adopted in this study, τ denotes the fixed time interval between successive releases of *Wolbachia*-infected mosquitoes. Seasonal infection-loss events occur at shifted impulsive times of the form (n+l)τ and, depending on the value of *l*, may coincide with the release instants. In real-world applications, the release size $u_n$ is naturally constrained by the limited availability of *Wolbachia*-infected mosquitoes. To reflect this practical limitation, we define the set of admissible release sizes as $U:= \{ 0 \le u_n\le u_{\text {max}}, \mid n \ge 0 \}$, where $u_{\text {max}} \ge 0$ denotes the maximum number of *Wolbachia*-carrying mosquitoes that can be introduced at any given intervention.

It is worth noting that temperature effects are incorporated in the present model exclusively through the infection-loss term φ(t), while the demographic parameters are assumed constant. This simplification allows the analytical treatment developed in the following sections, although temperature may also affect mosquito life-history traits such as egg viability, development, and adult survival. To evaluate the impact of this assumption, additional numerical simulations incorporating temperature-dependent demographic parameters are presented in Sect. 4.

Despite the simplifying assumptions adopted in the model, the proposed impulsive framework captures two key features of *Wolbachia*-based population replacement programs: periodic releases of infected mosquitoes and temperature-induced infection loss. This formulation provides a tractable framework for investigating how release strategies interact with environmental stress while preserving the essential biological mechanisms governing *Wolbachia* establishment and persistence.

### Model Behavior

#### Proposition 3

Let $(S_1(0), S_2(0))$ be a non-negative initial condition, $u_n \in U$, and $\varphi (t) \in [0,1]$. Let $(S_1(t), S_2(t))$ denote the solution of system (5a)–(5c). Then, $(S_1(t), S_2(t))$ remains non-negative for all t≥0.

#### Proof

Between impulses, the solution $(S_1(t), S_2(t))$ remains non-negative for all t≥0, as established in Theorem ()-(I).

At impulse instants of the type t=(n+l)τ, redistribution between the variables occurs, given by

$$ S_1(t^+) = S_1(t) + \varphi (t) S_2(t), \qquad S_2(t^+) = (1 - \varphi (t)) S_2(t). $$

Since $\varphi (t) \in [0,1]$ and $S_2(t) \ge 0$, both $S_1(t^+)$ and $S_2(t^+)$ remain non-negative.

At impulse instants of the type t=(n+1)τ, we have

$$ S_1(t^+) = S_1(t), \qquad S_2(t^+) = S_2(t) + u_n. $$

Since $S_1(t) \ge 0$, $S_2(t) \ge 0$, and $u_n \ge 0$ by hypothesis, it follows that $S_1(t^+), S_2(t^+) \ge 0$.

Therefore, by induction over all impulse events and continuous intervals, the non-negativity of $S_1(t)$ and $S_2(t)$ is preserved for all t≥0. □

#### Proposition 4

For each non-negative initial condition $(S_1(0), S_2(0))$, release amounts $u_n \in U$ ($u_n \ge 0$) at t=(n+1)τ, and transfers φ(t) defined by (4) at t=(n+l)τ, the system (5a)–(5c) admits a unique solution defined on [0,∞).

#### Proof

From Theorem (), the solution exists and is unique between impulses.

At discrete instants of the type t=(n+l)τ, the functions $S_1(t)$ and $S_2(t)$ admit well-defined right-hand limits:

$$ S_1(k\tau ^+) = \lim _{\epsilon \rightarrow 0^+} S_1(k\tau + \epsilon ), \qquad S_2(k\tau ^+) = \lim _{\epsilon \rightarrow 0^+} S_2(k\tau + \epsilon ). $$

At these moments, the impulsive transfers are given by

$$ S_1(t^+) = S_1(t) + \varphi (t) S_2(t), \qquad S_2(t^+) = (1 - \varphi (t)) S_2(t), $$

where φ(t) is defined by (4) and satisfies $\varphi (t) \in [0,1]$. These relations are linear and well defined, ensuring piecewise continuity of the solution and preservation of uniqueness after each impulse.

At instants of the type t=(n+1)τ, the impulsive releases occur according to

$$ S_1(t^+) = S_1(t), \qquad S_2(t^+) = S_2(t) + u_n, $$

with $u_n \in U$ and $u_n \ge 0$. This update is deterministic and also preserves the uniqueness of the solution.

Hence, the solutions are piecewise continuous, with well-defined left and right limits at every impulse time. By induction over the intervals $[n\tau , (n+1)\tau ]$, the solution can be uniquely and consistently extended to the entire domain [0,∞). □

For the analysis of the model with impulsive interventions, we define the following auxiliary system. This system is adjustable, since both the releases $u_n \in U$ and the intervention period τ can be adjusted according to the chosen intervention strategy. Our approach is to derive an analytical solution by assuming the simplification $S_1(t) \approx 0$. Consequently, the system below is obtained from Eqs. (5a)–(5c) under the condition $S_1(t) = 0$:

6 $$\begin{aligned} {\left\{ \begin{array}{ll} \dfrac{dB}{dt}(t) = B\psi _2 \left( 1 - \dfrac{B}{K} \right) - \delta _2 B, \quad t \ne (n + l)\tau , \text  t \ne (n + 1)\tau ,\\ B(t^+) = (1 - \varphi (t)) B(t), \qquad t = (n + l)\tau ,\\ B(t^+) = B(t) + u_n, \qquad t = (n + 1)\tau . \end{array}\right. } \end{aligned}$$

In what follows, we show the existence of a unique positive τ-periodic solution for system (6), which characterizes the long-term periodic dynamics of the system under impulsive interventions. For convenience, we define $r_2:=\psi _2 - \delta _2$ and $\bar{K}:= \frac{Kr_2}{\psi _2}.$

#### Theorem 5

Consider the auxiliary system (6), with $\varphi (t) \in [0,1]$ defined by (4) and $u_n \in U$. Then, there exists a unique positive τ-periodic solution $\bar{B}(t)$ given by:

7 $$\begin{aligned} \bar{B}(t) = {\left\{ \begin{array}{ll} \dfrac{\bar{K} B^+e^{r_2 (t-n\tau )}}{B^+\left( e^{r_2 (t-n\tau )} -1\right) +\bar{K}}, & n\tau< t \le (n+l)\tau ,\\ \dfrac{\bar{K} B^* e^{r_2 (t-(n+l)\tau )}}{B^*\left( e^{r_2 (t-(n+l)\tau )} -1\right) +\bar{K}}, & (n+l)\tau < t \le (n+1)\tau , \end{array}\right. } \end{aligned}$$

where

8 $$\begin{aligned} {\left\{ \begin{array}{ll} B^{+} \;=\; \dfrac{\bar{K}\big ((1-\varphi )e^{r_2\tau } - 1\big ) + u_n A + \sqrt{\big (\bar{K}\big ((1-\varphi )e^{r_2\tau } - 1\big ) + u_n A\big )^2 + 4 u_n \bar{K} A}}{2A},\\ B^* = \dfrac{(1-\varphi )\bar{K}B^+e^{r_2l\tau }}{B^+\left( e^{r_2l\tau }-1\right) +\bar{K}}, \end{array}\right. } \end{aligned}$$

with *A* defined in (23).

Moreover, the solution $\bar{B}(t)$ is globally asymptotically stable.

The proof of Theorem 5 is given in the Appendix C.

Establishing the uniform boundedness of the solutions is a crucial step in our analysis. Since we are dealing with population dynamics, it is natural to require that the trajectories remain biologically meaningful, that is, confined to a finite range for all time. Moreover, this property plays a central role in proving the global stability of the desired solutions, as it prevents the system from exhibiting unrealistic or uncontrolled behavior. Thus, after constructing and analyzing the auxiliary system, we now proceed to the proposition that ensures the uniform boundedness of the solutions of the impulsive model.

#### Proposition 6

Let $(S_1(t),S_2(t))$ be a solution of system (5a)-(5c), with positive parameters, $\varphi (t) \in [0,1]$, $u_n \in U$ and non-negative initial conditions. Then $(S_1(t),S_2(t))$ is uniformly bounded.

#### Proof

From Theorem 1-(III) we know that the solutions of the continuous part of the model are uniformly bounded. In particular, there exists a constant $M_c>0$ such that $S(t^-)=S_1(t^-)+S_2(t^-)\le M_c$ for every interval between impulses and for every solution.

We now show that the impulsive effects do not destroy this boundedness. Consider first the impulses occurring at times t=(n+l)τ. At such instants,

$$ S_1(t^+) = S_1(t^-) + \varphi (t) S_2(t^-), \qquad S_2(t^+) = (1-\varphi (t)) S_2(t^-), $$

with $$0 \le \varphi (t) \le 1$$. Hence,

$$ S(t^+) = S_1(t^+) + S_2(t^+) = S_1(t^-) + \varphi S_2(t^-) + (1-\varphi ) S_2(t^-) = S(t^-). $$

Thus, impulses of this type preserve *S* and do not increase its value.

Next, consider the impulses at times t=(n+1)τ. In this case,

$$ S_1(t^+) = S_1(t^-), \qquad S_2(t^+) = S_2(t^-) + u_n, $$

so that

$$ S(t^+) = S(t^-) + u_n. $$

By assumption, the release magnitudes satisfy $u_n \le u_{\max }$ for each *n*. Therefore,

$$ S(t^+) \le M_c + u_{\max }. $$

Between two consecutive impulses, the solution evolves according to the continuous dynamics and thus remains uniformly bounded. Consequently, defining

$$ M:= M_c + u_{\max }, $$

we obtain S(t)≤M for all t≥0 and for every solution of the full impulsive system. Hence both $S_1(t)$ and $S_2(t)$ remain uniformly bounded.

Finally, since the vector field of the continuous dynamics is locally Lipschitz and the jump maps either preserve *S* or map bounded sets into bounded sets, no finite-time blow-up can occur. Therefore, the solutions of the complete system is uniformly bounded. □

Having described the qualitative behavior of the model and the mechanisms governing the interaction between the two species, we now move to the formal analysis of the relevant equilibria in the impulsive regime. In particular, we examine the existence and stability of periodic solutions associated with the Wild-free state, in which the wild population of infected mosquitoes is eliminated, as well as the behavior of solutions that remain close to this state when small redistributive perturbations are introduced, namely, the impulses modeling the loss of infection due to high temperatures. In the next section, we establish conditions ensuring the existence of a positive, periodic, Wild-free solution and show that such a solution remains stable even in the presence of sufficiently small redistributive impulses. These results provide the necessary foundation for understanding how the system responds to perturbations and support the analysis of global stability around the dynamics we aim to achieve.

### Global Stability of Solutions

In this section, we investigate the existence and stability of the periodic solutions that constitute the main focus of this work, particularly the wild-mosquito-free solution, which represents the desired state in which the wild mosquito population is eliminated while the population of *Wolbachia*-infected mosquitoes remains periodically stable. We first establish the existence of this periodic solution for the unperturbed system and derive a sufficient condition ensuring its local stability through Floquet theory. We then show that this solution persists under sufficiently small redistributive impulses by means of the Implicit Function Theorem. Finally, we prove that the wild-mosquito-free periodic solution is globally asymptotically stable in the absence of redistributive impulses and that the perturbed periodic solution remains asymptotically stable for sufficiently small values of $\varphi _{\max }$.

From a biological perspective, establishing the existence and stability of these solutions is essential for assessing the feasibility of population management strategies. The wild-mosquito-free solution corresponds to the scenario in which the wild mosquito population is suppressed while the population of *Wolbachia*-infected mosquitoes persists over time, one of the central goals of this study. Demonstrating that this state not only exists but also remains stable under small seasonal perturbations indicates that moderate environmental fluctuations, seasonal variations, and limited redistributive events do not compromise the long-term suppression of the wild mosquito population. Thus, these theoretical results provide mathematical support for the robustness of the proposed control strategy under biologically realistic intervention scenarios.

#### Theorem 7

Let $(S_1(0),S_2(0))$ be non-negative initial conditions. Assume that, for $\varphi _{\max }=0$, system (5a)–(5c) admits the unique positive τ-periodic wild-mosquito-free solution $(0,\overline{S}_2(t))$.

Define

$$ \hat{K}:= K\left( 1-\frac{\delta _1}{\psi _1}\right) . $$

If $\overline{S}_2(t)>\hat{K},$ $t\in [0,\tau ],$ then there exists ε>0 such that, for every $0\le \varphi _{\max }<\varepsilon$, system (5a)–(5c) admits a positive τ-periodic solution $\bigl (S_1^\varphi (t),\overline{S}_2^\varphi (t)\bigr )$ in a neighborhood of $(0,\overline{S}_2(t))$. Moreover,

$$ \bigl (S_1^\varphi (t),\overline{S}_2^\varphi (t)\bigr ) \longrightarrow \bigl (0,\overline{S}_2(t)\bigr ) $$

uniformly on [0,τ] as $\varphi _{\max }\rightarrow 0$.

#### Proof

When $\varphi _{\max }=0$, we have $\varphi (t)\equiv 0$ for all t≥0. In this case, setting $S_1(t)\equiv 0$ in system (5a)–(5c) reduces the dynamics of $S_2$ to the auxiliary impulsive system (6). By Theorem 5, system (6) admits a unique positive τ-periodic solution $\overline{B}(t)$.

Therefore, the full system admits the wild-mosquito-free τ-periodic solution

$$ (0,\overline{S}_2(t)), \qquad \text {with } \overline{S}_2(t)=\overline{B}(t). $$

To establish the persistence of this periodic solution under small seasonal perturbations, we study the dynamics of small perturbations around the orbit and apply Floquet theory together with the Implicit Function Theorem.

Consider the perturbation variables

$$ S_1(t)=p(t), \qquad S_2(t)=\overline{S}_2(t)+q(t). $$

Substituting these expressions into system (5a)–(5c) and neglecting higher-order terms yields the variational system

$$\begin{aligned} \frac{d}{dt} \begin{pmatrix} p(t)\\ q(t) \end{pmatrix} = A(t) \begin{pmatrix} p(t)\\ q(t) \end{pmatrix}, \end{aligned}$$

where

$$\begin{aligned} A(t)=Df\bigl (0,\overline{S}_2(t)\bigr ). \end{aligned}$$

Using the Jacobian matrix computed in (14), we obtain

$$\begin{aligned} A(t)= \begin{pmatrix} \psi _1(1-\gamma ) \left( 1-\dfrac{\overline{S}_2(t)}{K} \right) -\delta _1 & 0 \\ -\dfrac{\psi _2\overline{S}_2(t)}{K} & \psi _2 \left( 1-\dfrac{2\overline{S}_2(t)}{K} \right) -\delta _2 \end{pmatrix}. \end{aligned}$$

Since $\overline{S}_2(t)$ is τ-periodic, the matrix *A*(*t*) is also τ-periodic. Therefore, Floquet theory can be applied.

The Jacobian matrices associated with the impulsive maps are

$$\begin{aligned} J_1= \begin{pmatrix} 1 & \varphi (t)\\ 0 & 1-\varphi (t) \end{pmatrix}, \qquad t=(n+l)\tau , \qquad J_2= \begin{pmatrix} 1 & 0\\ 0 & 1 \end{pmatrix}, \qquad t=(n+1)\tau . \end{aligned}$$

Since the reference periodic orbit is obtained for $\varphi _{\max }=0$, we have $\varphi (t)\equiv 0$ and therefore

$$\begin{aligned} J_1=J_2=I. \end{aligned}$$

Let Φ(t) denote the fundamental matrix solution of

$$\begin{aligned} \dot{\Phi }(t) = A(t)\Phi (t), \qquad \Phi (0)=I. \end{aligned}$$

For impulsive periodic systems, the monodromy matrix is obtained by composing the continuous flow with the Jacobian matrices associated with the impulses. Thus,

$$\begin{aligned} M= J_2 \Phi ((1-l)\tau ) J_1 \Phi (l\tau ). \end{aligned}$$

Since $J_1=J_2=I$, it follows that

$$\begin{aligned} M=\Phi (\tau ) = \begin{pmatrix} \mu _1 & 0\\ m_{21} & \mu _2 \end{pmatrix}, \qquad \begin{aligned} \mu _1&= \exp \!\left( \int _0^\tau \left[ \psi _1(1-\gamma ) \left( 1-\frac{\overline{S}_2(t)}{K} \right) -\delta _1 \right] dt \right) ,\\ \mu _2&= \exp \!\left( \int _0^\tau \left[ \psi _2 \left( 1-\frac{2\overline{S}_2(t)}{K} \right) -\delta _2 \right] dt \right) . \end{aligned} \end{aligned}$$

According to Floquet theory (Bainov and Simeonov 1993)^1^, since M is lower triangular, its spectrum is given by

$$ \sigma (M)=\{\mu _1,\mu _2\}. $$

Hence, $\mu _1$ and $\mu _2$ are the Floquet multipliers associated with the periodic solution $(0,\overline{S}_2(t))$.

The purpose of computing these multipliers is to verify the hyperbolicity of the periodic orbit, which is the fundamental requirement for its continuation under small perturbations of $\varphi _{\max }$.

By Theorem 5, the fixed point $B^+$ of the stroboscopic map is globally asymptotically stable. Therefore, perturbations in the $S_2$-direction decay after each period, and consequently,

$$ |\mu _2|<1. $$

Moreover, since

$$ \mu _1= \exp \left( \int _0^\tau \left[ \psi _1(1-\gamma ) \left( 1-\frac{\overline{S}_2(t)}{K} \right) -\delta _1 \right] dt \right) , $$

it follows that $0<\mu _1<1$ whenever

$$ \int _0^\tau \left[ \psi _1(1-\gamma ) \left( 1-\frac{\overline{S}_2(t)}{K} \right) -\delta _1 \right] dt <0. $$

Since $$0\le \gamma \le 1$$,

$$ \psi _1(1-\gamma ) \left( 1-\frac{\overline{S}_2(t)}{K} \right) -\delta _1 \le \psi _1 \left( 1-\frac{\overline{S}_2(t)}{K} \right) -\delta _1. $$

Thus, it is sufficient to require that

$$ \psi _1 \left( 1-\frac{\overline{S}_2(t)}{K} \right) -\delta _1 <0, $$

for all $t\in [0,\tau ]$. Equivalently,

$$ \overline{S}_2(t)> K\left( 1-\frac{\delta _1}{\psi _1} \right) =\hat{K}. $$

Under this sufficient condition,

$$ 0<\mu _1<1. $$

Since $|\mu _2|<1$ and $0<\mu _1<1$, no Floquet multiplier is equal to one. Hence, the periodic orbit $(0,\overline{S}_2(t))$ is hyperbolic.

To continue this periodic orbit for small values of $\varphi _{\max }$, consider the Poincaré map $P_{\varphi _{\max }}(X_0):=X(\tau ^+;X_0,\varphi _{\max })$, where $X(t;X_0,\varphi _{\max })$ denotes the solution of the impulsive system with initial condition $X_0$.

The periodic orbit $(0,\overline{S}_2(t))$ corresponds to the fixed point $X^*=(0,B^+)$ of the unperturbed map $P_0$, satisfying $P_0(X^*)=X^*$.

Define $F(X,\varphi _{\max })=P_{\varphi _{\max }}(X)-X$. Then $F(X^*,0)=0$ and, since the derivative of the Poincaré map at a periodic orbit coincides with the monodromy matrix,

$$\begin{aligned} D_XF(X^*,0) = DP_0(X^*)-I = M-I = \begin{pmatrix} \mu _1-1 & 0\\ m_{21} & \mu _2-1 \end{pmatrix}. \end{aligned}$$

Therefore,

$$\begin{aligned} \det \bigl (D_XF(X^*,0)\bigr ) = (\mu _1-1)(\mu _2-1). \end{aligned}$$

Since $\mu _1\ne 1$ and $\mu _2\ne 1$, we obtain $\det (D_XF(X^*,0))\ne 0$, and hence $D_XF(X^*,0)$ is invertible.

The Implicit Function Theorem then guarantees the existence of ε>0 and a continuous branch of fixed points $X(\varphi _{\max })$, defined for $0\le \varphi _{\max }<\varepsilon$, such that

$$\begin{aligned} P_{\varphi _{\max }} \bigl (X(\varphi _{\max })\bigr ) = X(\varphi _{\max }). \end{aligned}$$

Since fixed points of the Poincaré map are in one-to-one correspondence with periodic solutions of the impulsive system, it follows that, for sufficiently small values of $\varphi _{\max }$, system (5a)–(5c) admits a positive τ-periodic solution $\bigl (S_1^{\varphi }(t),\overline{S}_2^{\varphi }(t)\bigr )$ in a neighborhood of $(0,\overline{S}_2(t))$.

Moreover,

$$\begin{aligned} \bigl ( S_1^{\varphi }(t), \overline{S}_2^{\varphi }(t) \bigr ) \longrightarrow \bigl ( 0, \overline{S}_2(t) \bigr ) \qquad \text {as } \varphi _{\max }\rightarrow 0. \end{aligned}$$

This completes the proof.

□

#### Theorem 8

(Global stability and stability under small perturbations)

Under the assumptions of Theorem 7, let $(0,\overline{S}_2(t))$ be the wild-mosquito-free τ-periodic solution of system (5a)–(5c) corresponding to $\varphi _{\max }=0$. Then:

(i) The periodic solution $(0,\overline{S}_2(t))$ is globally asymptotically stable in $\mathbb R_+^2$.
(ii) Let $(S_1^\varphi (t),\overline{S}_2^\varphi (t))$ be the periodic solution provided by Theorem 7. Then, for sufficiently small values of $\varphi _{\max }$, this periodic solution is asymptotically stable. Moreover,
  $$ S_1^\varphi (t)\rightarrow 0 $$
  uniformly as $\varphi _{\max }\rightarrow 0$, and
  $$ \overline{S}_2^\varphi (t) \longrightarrow \overline{S}_2(t) $$
  uniformly on [0,τ].

#### Proof

*(i) Global stability of the wild-mosquito-free periodic solution.*

By Theorem 7, the Floquet multipliers associated with the periodic solution

$$ (0,\overline{S}_2(t)) $$

satisfy

$$ |\mu _1|<1, \qquad |\mu _2|<1. $$

Hence, by Floquet theory, the periodic solution $(0,\overline{S}_2(t))$ is locally asymptotically stable. It remains to prove its global attractivity.

From the second equation of system (5a)–(5c), we obtain

$$ \frac{dS_2}{dt} \le \psi _2\left( 1-\frac{S_2}{K}\right) S_2-\delta _2S_2. $$

Thus, by the Comparison Theorem (see Lakshmikantham et al. (1989); Alves et al. (2026)), $S_2(t)$ can be compared with the auxiliary impulsive system (6). By Theorem 5,

$$ B(t)\longrightarrow \overline{B}(t), \qquad t\rightarrow \infty . $$

Hence, for every ε>0, there exists $t_1>0$ such that

$$ S_2(t)\le B(t)<\overline{B}(t)+\varepsilon , \qquad t>t_1. $$

Moreover,

$$ \frac{S_1+(1-\gamma )S_2}{S_1+S_2}\le 1, $$

which implies

$$ \frac{dS_1}{dt} \le S_1\psi _1 \left( 1-\frac{S_1+S_2}{K} \right) -\delta _1S_1. $$

Motivated by the sufficient condition derived in the proof of Theorem 7, consider the following auxiliary comparison system:

9 $$\begin{aligned} {\left\{ \begin{array}{ll} \dfrac{dZ_1}{dt} = Z_1\psi _1 \left( 1-\dfrac{Z_1+\hat{K}}{K} \right) -\delta _1Z_1, & t\ne (n+l)\tau ,\;(n+1)\tau ,\\ Z_1(t^+)=Z_1(t), & t=(n+l)\tau ,\;(n+1)\tau ,\\ Z_1(0)=S_1(0), \end{array}\right. } \end{aligned}$$

where

$$ \hat{K} = K\left( 1-\frac{\delta _1}{\psi _1}\right) . $$

By construction, the zero solution of system (9) is globally asymptotically stable. Therefore,

$$ Z_1(t)\longrightarrow 0, \qquad t\rightarrow \infty . $$

By the Comparison Theorem,

$$ S_1(t)\le Z_1(t), $$

and therefore

$$ S_1(t)\longrightarrow 0, \qquad \text {as }t\rightarrow \infty . $$

It follows that, for every ε>0, there exists $t_2>0$ such that

$$ 0\le S_1(t)<\varepsilon , \qquad t>t_2. $$

Substituting this estimate into the second equation of system (5a)–(5c), we obtain

$$ \frac{dS_2}{dt} \ge \psi _2S_2 \left( 1-\frac{\varepsilon +S_2}{K} \right) -\delta _2S_2, \qquad t>t_2. $$

Consider the following auxiliary impulsive system:

10 $$\begin{aligned} {\left\{ \begin{array}{ll} \dfrac{dW}{dt} = \psi _2W \left( 1-\dfrac{\varepsilon +W}{K} \right) -\delta _2W, & t\ne (n+l)\tau ,\;(n+1)\tau ,\\ W(t^+) = (1-\varphi (t))W(t), & t=(n+l)\tau ,\\ W(t^+) = W(t)+u_n, & t=(n+1)\tau ,\\ W(t_2)=S_2(t_2). \end{array}\right. } \end{aligned}$$

By the Comparison Theorem,

$$ W(t)\le S_2(t), \qquad t>t_2. $$

Proceeding as in the proof of Theorem 5, replacing the logistic term by $1-\frac{\varepsilon +W}{K},$ system (10) admits a positive τ-periodic solution $\overline{W}_{\varepsilon }(t)$ satisfying

$$ W(t)\longrightarrow \overline{W}_{\varepsilon }(t), \qquad \text {as }t\rightarrow \infty . $$

Moreover, since the coefficients of the system depend continuously on ε, we have

$$ \overline{W}_{\varepsilon }(t) \longrightarrow \overline{B}(t) $$

uniformly on [0,τ] as $\varepsilon \rightarrow 0$. Hence, for ε>0 sufficiently small,

$$ \overline{B}(t)-\varepsilon \le \overline{W}_{\varepsilon }(t) \le S_2(t) \le \overline{B}(t)+\varepsilon , $$

for all sufficiently large *t*. Letting $\varepsilon \rightarrow 0$, we conclude that

$$ S_2(t)\longrightarrow \overline{B}(t)=\overline{S}_2(t). $$

Since

$$ S_1(t)\longrightarrow 0, $$

it follows that

$$ (S_1(t),S_2(t)) \longrightarrow (0,\overline{S}_2(t)), \qquad \text {as }t\rightarrow \infty . $$

Therefore, the periodic solution $(0,\overline{S}_2(t))$ is globally asymptotically stable in $\mathbb {R}_+^2$.

*(ii) Stability under small seasonal perturbations.*

By Theorem 7, for sufficiently small values of $\varphi _{\max }$, system (5a)–(5c) admits a positive τ-periodic solution

$$ (S_1^\varphi (t),\overline{S}_2^\varphi (t)), $$

which converges uniformly to

$$ (0,\overline{S}_2(t)) $$

on [0,τ] as $\varphi _{\max }\rightarrow 0$. Moreover, by Theorem 7, the Floquet multipliers associated with the unperturbed periodic solution satisfy

$$ |\mu _1|<1, \qquad |\mu _2|<1. $$

Since the vector field and the impulsive maps depend smoothly on the parameter $\varphi _{\max }$, the corresponding Floquet multipliers vary continuously with $\varphi _{\max }$. Hence, for sufficiently small values of $\varphi _{\max }$, all Floquet multipliers associated with

$$ (S_1^\varphi (t),\overline{S}_2^\varphi (t)) $$

remain strictly inside the unit circle.

Therefore, by Floquet theory, the periodic solution

$$ (S_1^\varphi (t),\overline{S}_2^\varphi (t)) $$

is locally asymptotically stable.

Finally,

$$ S_1^\varphi (t)\rightarrow 0,\qquad \overline{S}_2^\varphi (t)\rightarrow \overline{S}_2(t), $$

uniformly on [0,τ] as $\varphi _{\max }\rightarrow 0$.

Hence, for all sufficiently small values of $\varphi _{\max }$, the periodic solution

$$ (S_1^\varphi (t),\overline{S}_2^\varphi (t)) $$

is asymptotically stable for all sufficiently small seasonal perturbations. □

Together, Theorems 7 and 8 establish the mathematical foundation of the proposed control strategy. Under condition $\overline{S}_2(t)>\hat{K},$ the wild-mosquito-free periodic solution exists, persists under sufficiently small redistributive impulses, and is globally asymptotically stable. Consequently, the wild mosquito population cannot invade, while the population of *Wolbachia*-infected mosquitoes remains periodically established. Moreover, the persistence and stability of this solution under small redistributive losses of infection, modeled by $\varphi _{\max }$, demonstrate the effectiveness of the proposed release strategy under biologically relevant perturbations.

### A Method for Selecting $u_n$: A Sufficient Condition

In this section, we exploit the condition $\overline{S}_2(t)>\hat{K},$ established in the proof of Theorem 7, to derive a lower bound for the release size $u_n$ ensuring the stability of the wild-mosquito-free periodic solution and the stability of the perturbed periodic solutions. More precisely, we determine a function $\eta (\tau )$ such that, whenever $u_n\ge \eta (\tau ),$ the sufficient condition is satisfied and, consequently, the conclusions of Theorems 7 and 8 hold.

To this end, we need to guarantee that $\bar{S}_2(t) > \hat{K}$ for all t≥0. It is sufficient to require that the minimum value attained by $\bar{S}_2(t)$ within a single release cycle is greater than $\hat{K}$. We denote this minimum value by

$$ \bar{S}_2^{\min } = \min \bar{S}_2(t), \quad t \in (n\tau , (n+1)\tau ]. $$

By examining the behavior of the solution over one period τ, which is defined piecewise as

$$\begin{aligned} \bar{S}_2(t) = {\left\{ \begin{array}{ll} \dfrac{\bar{K} B^+ e^{r_2 (t-n\tau )}}{B^+\left( e^{r_2 (t-n\tau )} - 1\right) + \bar{K}}, & n\tau< t \le (n+l)\tau ,\\ \dfrac{\bar{K} B^* e^{r_2 (t-(n+l)\tau )}}{B^*\left( e^{r_2 (t-(n+l)\tau )} - 1\right) + \bar{K}}, & (n+l)\tau < t \le (n+1)\tau , \end{array}\right. } \end{aligned}$$

where $B^+$ and $B^*$ are given in (8), we find that the minimum value $\bar{S}_2^{\min }$ is attained at time t=(n+l)τ. Therefore,

$$ \bar{S}_2((n+l)\tau ) = \bar{S}_2^{\min } = B^*. $$

#### Derivation of the Sufficient Condition

Since the minimum value of $\overline{S}_2(t)$ over each period is given by $B^*$, imposing the threshold condition

$$ B^* > \hat{K} $$

and performing algebraic manipulations (similar to Alves et al. (2026)) leads to a sufficient requirement on the release magnitude:

$$ u_n \ge \eta (\tau ), \qquad \tau > 0, $$

where the function $\eta (\tau )$ is

11 $$\begin{aligned} \begin{aligned} \eta (\tau )&= \phi (\tau )\left( \dfrac{ \phi (\tau )A -\bar{K}\big (e^{r_2\tau }(1-\varphi )-1\big ) }{ \bar{K}+A\phi (\tau ) } \right) ,\\ \phi (\tau )&= \frac{ \bar{K}\hat{K} }{ \bar{K}(1-\varphi )e^{r_2l\tau } -\hat{K}\big (e^{r_2l\tau }-1\big ) }. \end{aligned} \end{aligned}$$

#### Establishing a Conservative Bound

The function $\eta (\tau )$ may take negative values for certain τ>0. Since biologically the release quantity must satisfy $u_n>0$, we restrict attention to values of τ for which $\eta (\tau )$ is positive. To obtain a uniform sufficient condition valid for all such τ, we introduce the conservative requirement

12 $$\begin{aligned} u_n> \max _{\tau>0} \eta (\tau ) > 0. \end{aligned}$$

We now show that this maximum indeed exists.

##### Proposition 9

Let $\eta :[0,+\infty )\rightarrow \mathbb {R}$ be the function defined in (11), with $\phi (\tau )$ and A(τ) as defined in (11) and (23), respectively. Let $\bar{K}$, $\hat{K}$, and $r_2$ be positive constants, l∈[0,1], and $\varphi \in [0,1)$.

Then, the function $\eta (\tau )$ is well-defined and continuous on [0,+∞), and admits at least one global maximum point $\tau ^*\in [0,+\infty )$.

##### Proof

The function $\phi (\tau )$ is defined as a quotient of exponential functions. Its denominator is given by

$$ D(\tau )=e^{r_2 l \tau }\big (\bar{K}(1-l)-\hat{K}\big )+\hat{K}, $$

which is continuous. Since $\bar{K},\hat{K}>0$ and $e^{r_2 l \tau }\ge 1$ for all $\tau \ge 0$, it follows that D(τ)>0 for all $\tau \ge 0$. Hence, $\phi (\tau )$ is continuous on [0,+∞).

Moreover, since the denominator of $\eta (\tau )$ does not vanish in this interval, we conclude that $\eta (\tau )$ is continuous on [0,+∞).

We now analyze the asymptotic behavior of $\eta (\tau )$ as $\tau \rightarrow +\infty$. We observe that

$$ \phi (\tau )\sim \frac{\hat{K}}{\bar{K}(1-l)-\hat{K}}\,e^{-r_2 l \tau }, $$

which implies that $\phi (\tau )\rightarrow 0$ as $\tau \rightarrow +\infty$.

Consider the term

$$ G(\tau )=\frac{\phi (\tau )A(\tau )-\bar{K}\big (e^{r_2\tau }(1-\varphi )-1\big )}{\bar{K}+A(\tau )\phi (\tau )}. $$

From the definition of A(τ), we have $A(\tau )=\mathcal {O}(e^{r_2\tau })$ as $\tau \rightarrow +\infty$. Since $\phi (\tau )=\mathcal {O}(e^{-r_2 l \tau })$, it follows that

$$ \phi (\tau )A(\tau )=\mathcal {O}(e^{r_2(1-l)\tau }). $$

Because l∈[0,1], this term grows at most like $e^{r_2\tau }$. Therefore, both the numerator and the denominator of G(τ) have at most exponential growth of order $e^{r_2\tau }$. Consequently, there exists a constant C>0 such that

$$ |G(\tau )|\le C\,e^{r_2\tau } $$

for τ sufficiently large.

Since $\phi (\tau )=\mathcal {O}(e^{-r_2 l \tau })$, we obtain

$$ \eta (\tau )=\phi (\tau )G(\tau )=\mathcal {O}(e^{-r_2 l \tau }), $$

which implies that

$$ \lim _{\tau \rightarrow +\infty }\eta (\tau )=0. $$

Moreover, evaluating at τ=0, we obtain

$$ \eta (0)=\frac{\hat{K}\,\varphi }{1-\varphi }\ge 0. $$

Thus, $\eta (\tau )$ is continuous on [0,+∞), nonnegative, and tends to zero at infinity.

If $\eta (\tau )$ is strictly decreasing, then the global maximum occurs at $\tau ^*=0$. Otherwise, if there exists τ>0 such that $\eta (\tau )>\eta (0)$, then the function initially increases and, since it tends to zero at infinity, it must attain a maximum at some point in the interval.

By the extension of the Weierstrass theorem to non-compact intervals namely, if a continuous function $f:[0,+\infty )\rightarrow \mathbb {R}$ is nonnegative and satisfies $\lim _{x\rightarrow +\infty }f(x)=0$, then *f* attains a global maximum it follows that there exists $\tau ^*\in [0,+\infty )$ such that

$$ \eta (\tau ^*)=\max _{\tau \ge 0}\eta (\tau ). $$

□

With these results, we establish a uniform threshold for the release of *Wolbachia*-infected mosquitoes, valid for any release period τ>0. For values of τ for which $\eta (\tau )$ is positive, the function $\eta (\tau )$ itself provides a sufficient release quantity. In cases where $\eta (\tau )$ becomes negative, we instead use the global maximum of η, whose existence is guaranteed by Proposition 9. This maximum acts as a conservative bound that applies uniformly for all τ>0.

Therefore, for different sets of biological parameters, possibly corresponding to different *Wolbachia* strains, the function $\eta (\tau )$ provides a conservative sufficient criterion for selecting the release size. Choosing $u_n\ge \eta (\tau )$ guarantees the sufficient condition established in Theorem 7 and, consequently, the existence and stability properties proved in Theorems 7 and 8. Thus, the analytical threshold establishes a direct connection between the mathematical analysis and the design of biologically feasible release strategies.

In the next section, we present numerical simulations illustrating the theoretical results obtained in this section. In particular, we compare different *Wolbachia* strains, investigate the influence of high temperatures on infection loss, and assess the conservativeness of the analytical threshold by considering release sizes below $\eta (\tau )$.

## Numerical Simulations

The numerical simulations presented in this section investigate the behavior of the three *Wolbachia* strains (wMelPop, wMel, and wAlbB) under temperature-dependent infection loss. Particular emphasis is placed on evaluating the proposed release strategy, comparing the long-term performance of the different strains, and examining whether the main analytical conclusions remain valid under biologically relevant extensions of the model.

Unless otherwise stated, releases are performed during the first 365 days and the system is subsequently simulated for an additional 730 days, resulting in a total simulation period of three years. The complete parameterization and simulation scenarios are summarized in Tables 2, 3, 4, 5, and 6. The numerical study is organized into three parts: dynamics during the release phase, long-term dynamics after the release program ends, and robustness analyses incorporating temperature-dependent demographic variation and imperfect maternal transmission.

**Table 2:** Multiplicative factors applied to the baseline demographic parameters of the wild mosquito population

	Multiplicative factor		
*Wolbachia* strain	Birth rate $\alpha _\psi$	Mortality rate $\alpha _\delta$	References
wMelPop	0.50	1.50	This study
wMel	0.95	1.10	(Xue et al. 2018)
wAlbB	0.85	1.00	(Xue et al. 2018)
Wild	1.00	1.00	(Xue et al. 2018)

The parameters for *Wolbachia*-infected mosquitoes are computed as $( \psi _2 = \alpha _\psi \psi _1 ) \text { and } ( \delta _2 = \alpha _\delta \delta _1, )$ where $\alpha _\psi$ and $\alpha _\delta$ are the strain-specific multiplicative factors listed in this table

The analysis focuses on how strain-dependent differences in heat tolerance, virus-blocking efficiency, and fitness effects on the host mosquito are incorporated into the model through multiplicative factors applied to the demographic parameters of the wild mosquito population. These factors, reported in Table 2, are derived from the biological characteristics summarized in Table 1 and play a central role in determining the establishment and persistence of *Wolbachia*-infected populations under temperature-induced infection loss.

The multiplicative factors associated with the wMel and wAlbB strains are taken from Xue et al. (2018). In contrast, no unified quantitative estimates are available for wMelPop within the present modeling framework. Therefore, the values $\alpha _\psi =0.50$ and $\alpha _\delta =1.50$ were selected to represent the higher fitness costs consistently reported for this strain, including reduced longevity and reproductive performance (Ritchie et al. 2015; Axford et al. 2016; Ross 2021; Orozco-Gonzales et al. 2024).

To examine the sensitivity of these conclusions to the assumed values of $\alpha _\psi$ and $\alpha _\delta$, we performed a local sensitivity analysis around the baseline values $\alpha _\psi =0.50$ and $\alpha _\delta =1.50$.

**Table 3:** Sensitivity of the analytical release thresholds for the wMelPop strain under variations of the multiplicative factors $\alpha _\psi$ and $\alpha _\delta$

Variation in $\alpha _\psi$ ($\alpha _\delta =1.50$)			Variation in $\alpha _\delta$ ($\alpha _\psi =0.50$)		
$\alpha _\psi$	η(7)	η(14)	$\alpha _\delta$	η(7)	η(14)
0.40	229.95	647.25	1.40	189.76	550.49
0.50	212.78	648.09	1.50	212.78	648.09
0.60	194.88	642.32	1.60	236.30	759.26

The analytical thresholds vary with the assumed demographic parameters. Increasing the birth-rate factor $\alpha _\psi$ generally decreases the required release threshold, whereas increasing the mortality-rate factor $\alpha _\delta$ produces the opposite effect, with the latter having a stronger influence, particularly for η(14). Despite these quantitative variations, the main comparative conclusion remains unchanged over the parameter ranges considered: wMelPop consistently requires substantially larger release amplitudes than wMel and wAlbB. Thus, within the ranges explored in the sensitivity analysis, this conclusion does not depend on the specific baseline values $\alpha _\psi =0.50$ and $\alpha _\delta =1.50$.

**Table 4:** Temperature-induced infection-loss scenarios considered in the numerical simulations

Scenario	wMelPop	wMel	wAlbB
Low infection loss	0.0015	0.0010	0.0005
Moderate infection loss	0.0150	0.0100	0.0050
High infection loss	0.1500	0.1000	0.0500

To investigate the effects of thermal stress on *Wolbachia* establishment and persistence, we consider three temperature-induced infection-loss scenarios, denoted as low, moderate, and high infection loss. The corresponding values of $\varphi _{\max }$ are reported in Table 4.

The values of $\varphi _{\max }$ are intended as exploratory scenario parameters rather than direct quantitative calibrations. They represent progressively stronger levels of temperature-induced infection loss while preserving the experimentally observed hierarchy of thermal sensitivity among the three strains. Consequently, the simulations provide a qualitative assessment of strain robustness under increasingly adverse thermal conditions rather than precise field-calibrated predictions.

**Table 5:** Common parameters used in the impulsive population-dynamic model

Description	Parameter	Value	Range
Relative timing of infection loss within the release period τ	*l*	0.5	[0,1]
Time of the year corresponding to the temperature peak	$t_{\text {peak}}$	180	–
Carrying capacity for the mosquito population	*K*	500	–

**Table 6:** The values of model parameters for the wild mosquito population and for mosquitoes infected with different *Wolbachia* strains

Description	Parameter	Value	Units	Range
Wild mosquitoes $S_1$				
Birth rate	$\psi _1$	0.300	day^-1^	–
Death rate	$\delta _1$	0.071	day^-1^	–
wMelPop-infected mosquitoes $S_2$				
Birth rate	$\psi _2$	0.150	day^-1^	–
Death rate	$\delta _2$	0.106	day^-1^	–
CI probability	γ	0.90	–	[0.9,1]
wMel-infected mosquitoes $S_2$				
Birth rate	$\psi _2$	0.285	day^-1^	–
Death rate	$\delta _2$	0.0781	day^-1^	–
CI probability	γ	0.98	–	[0.98,1]
wAlbB-infected mosquitoes $S_2$				
Birth rate	$\psi _2$	0.255	day^-1^	–
Death rate	$\delta _2$	0.071	day^-1^	–
CI probability	γ	0.90	–	[0.9,1]

Tables 5 and 6 summarize the remaining parameters used throughout the numerical experiments. Common parameters are listed in Table 5, whereas Table 6 reports the strain-specific demographic and cytoplasmic incompatibility parameters obtained from the multiplicative factors in Table 2. Together, these parameters define the baseline configurations adopted throughout the numerical study. The numerical results obtained under these scenarios are presented in the following subsections.

### Dynamics During the Release Phase

In this section, we investigate the practical implications of the analytical release threshold $\eta (\tau )$ derived in Sect. 3. Since $\eta (\tau )$ provides a sufficient condition for the establishment of *Wolbachia*-infected mosquitoes, we assess its conservativeness by comparing simulations performed with the theoretical release amplitude $\eta (\tau )$ and a reduced release amplitude equal to $0.25\eta (\tau )$. The analysis is carried out under low and moderate temperature-induced infection loss and for release intervals of τ=7 and τ=14 days.

#### Case 1: Low Infection Loss

We now consider low infection-loss intensities, with $\varphi _{\max }=0.0015$, 0.001, and 0.0005 for the wMelPop, wMel, and wAlbB strains, respectively. This scenario allows us to investigate the behavior of the analytical threshold under favorable environmental conditions, where infection loss remains relatively weak.

***Subcase 1.1:*** τ=7

**Fig. 3:**
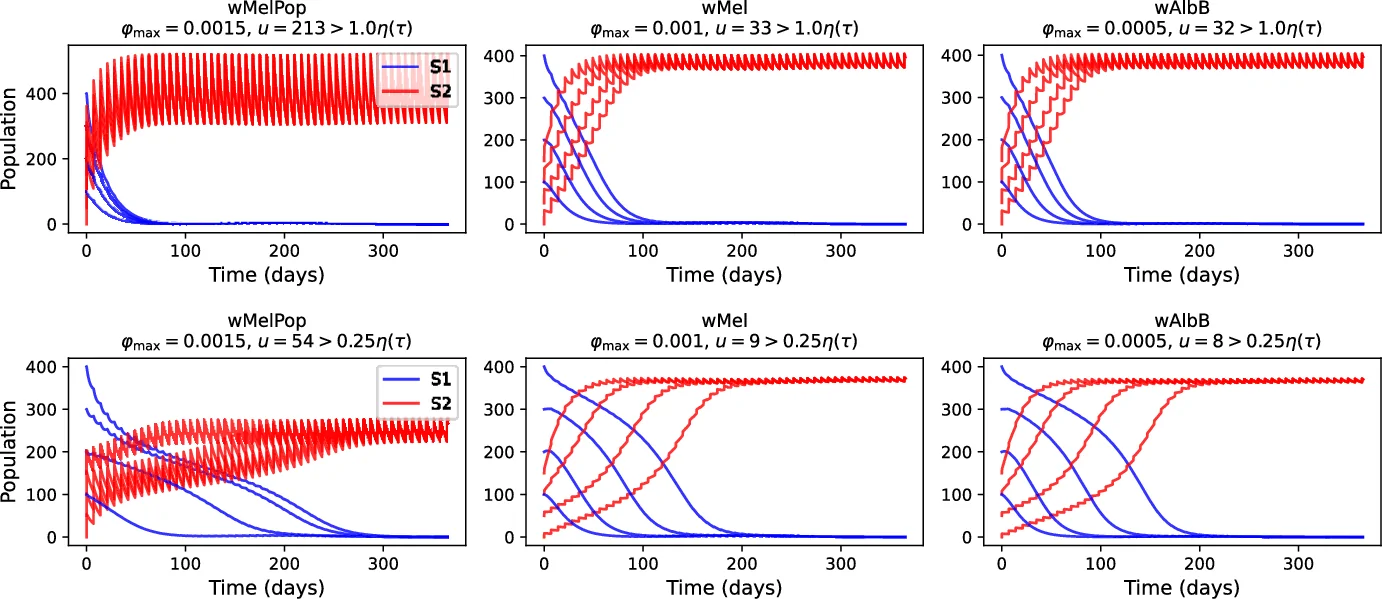
Population dynamics of wild and *Wolbachia*-infected mosquitoes under low infection loss and release interval τ=7, simulated under four distinct initial conditions: (400, 0), (300, 50), (200, 100), and (100, 150). The first row corresponds to the analytical release amplitude $u_n=\eta (7)$, while the second row corresponds to the reduced release amplitude $u_n=0.25\eta (7)$ (Color figure online)

Figure 3 compares the population dynamics obtained with the analytical release amplitude $u_n=\eta (7)$ and the reduced release amplitude $u_n=0.25\eta (7)$ under low infection-loss intensities. Successful establishment of the infected population is observed for all three strains and for all initial conditions considered, regardless of the release amplitude. When the analytical threshold is used, population replacement occurs rapidly, with the wild population declining to extinction within a relatively short time interval.

Reducing the release amplitude to one quarter of the analytical threshold does not prevent establishment, but it noticeably alters the transient dynamics. This effect is particularly pronounced for the wMelPop strain, which exhibits a substantially longer coexistence phase, larger oscillations, and a slower suppression of the wild population. By contrast, the wMel and wAlbB strains remain highly stable, reaching infected-dominated regimes with only a moderate increase in convergence time. These results further indicate that the analytical threshold $\eta (\tau )$ is a conservative sufficient condition and that, under low infection-loss intensities, successful invasion may occur with release amplitudes considerably smaller than the theoretical bound.

***Subcase 1.2:*** τ=14

**Fig. 4:**
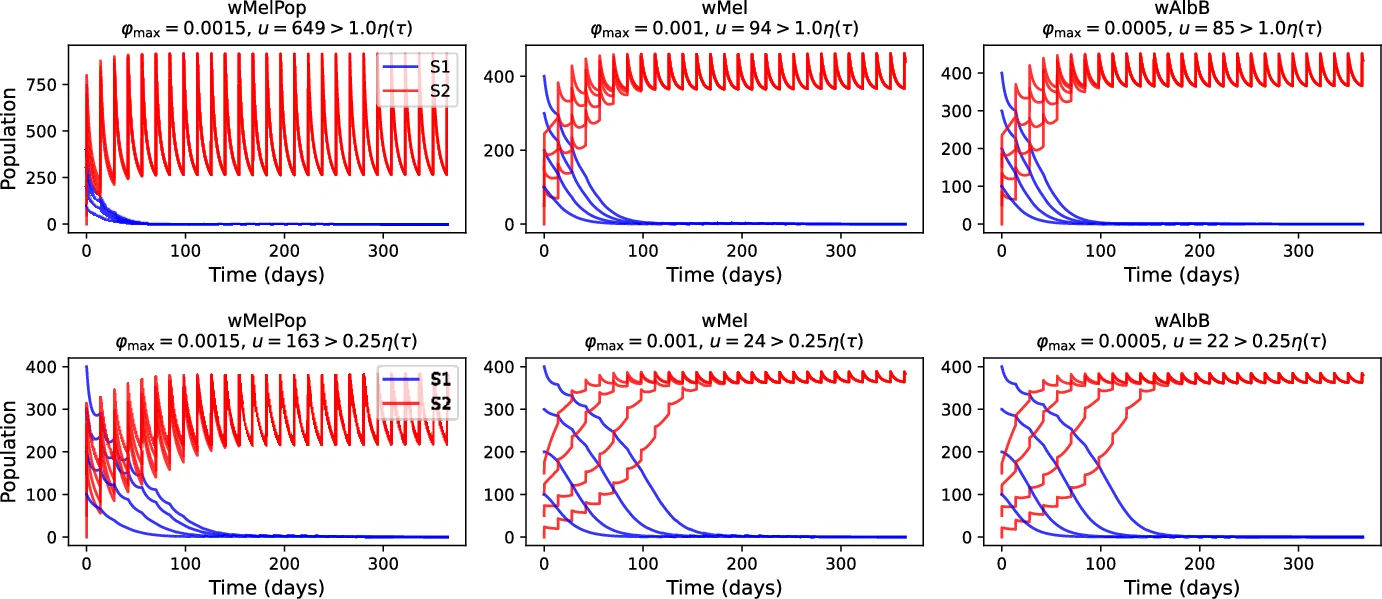
Population dynamics of wild and *Wolbachia*-infected mosquitoes under low infection loss and release interval τ=14, simulated under four distinct initial conditions: (400, 0), (300, 50), (200, 100), and (100, 150). The first row corresponds to the analytical release amplitude $u_n=\eta (14)$, while the second row corresponds to the reduced release amplitude $u_n=0.25\eta (14)$ (Color figure online)

Figure 4 presents the analogous comparison for the longer release interval τ=14. As in the case τ=7, successful establishment is observed for all strains and all initial conditions under both release amplitudes. Although reducing the release effort does not affect the long-term outcome, it leads to slower transient dynamics, particularly for the wMelPop strain. Nevertheless, successful establishment is still achieved under only 25% of the analytical threshold, even with the longer interval between releases.

Taken together, these results indicate that low infection-loss intensities substantially increase the robustness of *Wolbachia* establishment. Successful invasion is observed even when the release amplitude is reduced to 25% of the analytical threshold, with the wMel and wAlbB strains being only mildly affected by the smaller release effort.

#### Case 2: Moderate Infection Loss

In this case, we consider moderate infection-loss intensities, namely $\varphi _{\max }=0.015$, 0.01, and 0.005 for the strains wMelPop, wMel, and wAlbB, respectively.

***Subcase 2.1:*** τ=7

**Fig. 5:**
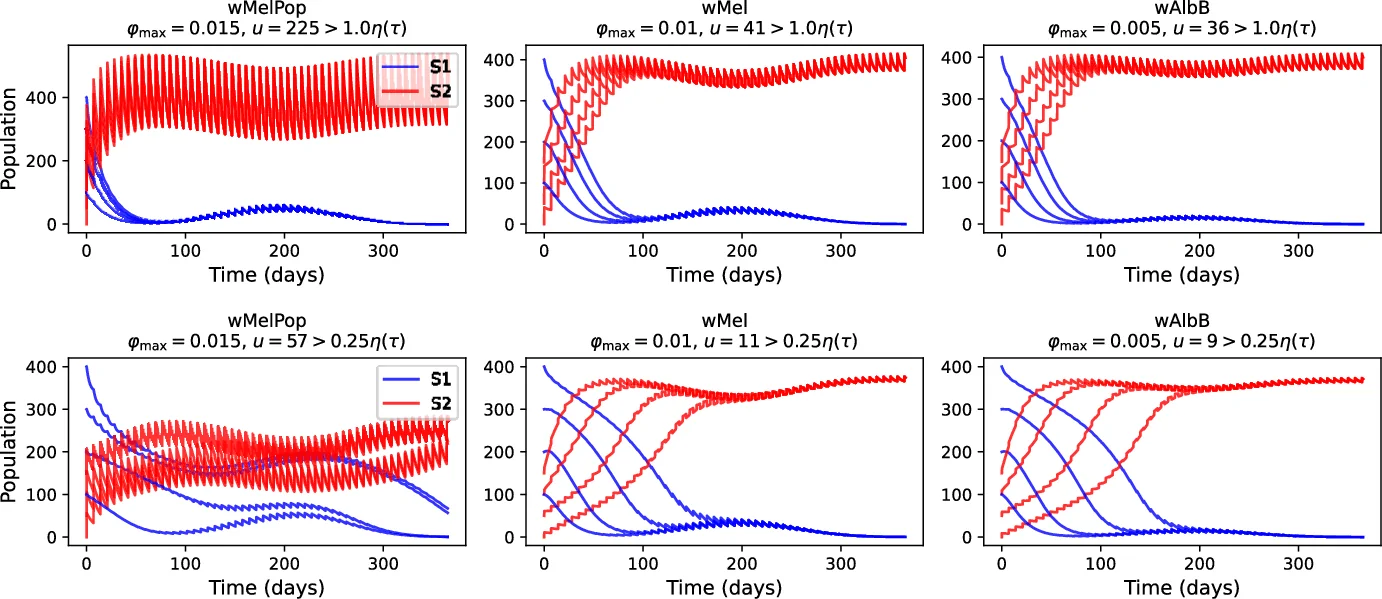
Population dynamics of wild and *Wolbachia*-infected mosquitoes under moderate infection loss and release interval τ=7, simulated under four distinct initial conditions: (400, 0), (300, 50), (200, 100), and (100, 150). The first row corresponds to the analytical release amplitude $u_n=\eta (7)$, while the second row corresponds to the reduced release amplitude $u_n=0.25\eta (7)$ (Color figure online)

Figure 5 compares the population dynamics obtained using the analytical release amplitude $u_n=\eta (7)$ and the reduced release amplitude $u_n=0.25\eta (7)$ under moderate infection-loss intensities.

For all three strains, successful establishment of the infected population is still observed when the release amplitude is reduced to one quarter of the analytical threshold. Nevertheless, the smaller release effort significantly affects the transient dynamics and the convergence rate toward an infected-dominated state.

This effect is particularly evident for the wMelPop strain, which exhibits larger oscillations, a prolonged coexistence phase, and a substantially slower suppression of the wild population. By contrast, the wMel and wAlbB strains remain comparatively stable, converging more rapidly to infected-dominated regimes. These results suggest that, although the analytical threshold $\eta (\tau )$ is conservative with respect to establishment, larger release amplitudes may substantially accelerate the replacement process and shorten the transient period before infected mosquitoes become dominant.

***Subcase 2.2:*** τ=14

**Fig. 6:**
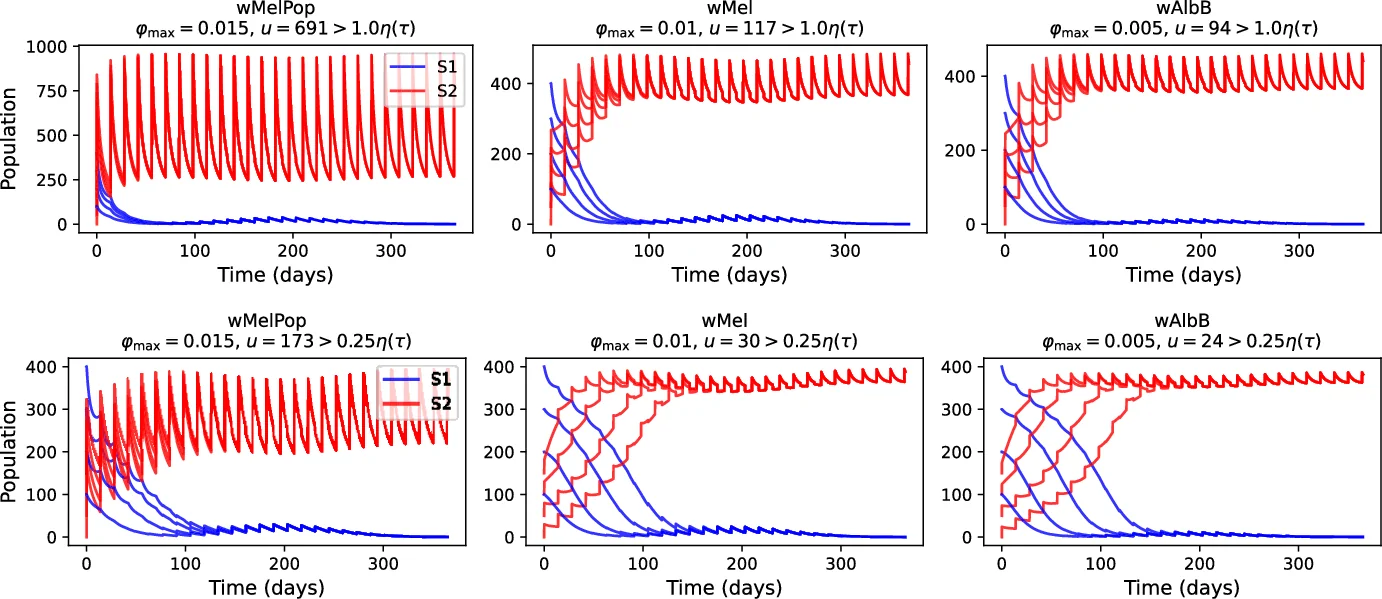
Population dynamics of wild and *Wolbachia*-infected mosquitoes under moderate infection loss and release interval τ=14, simulated under four distinct initial conditions: (400, 0), (300, 50), (200, 100), and (100, 150). The upper panels correspond to the analytical release amplitude $u_n=\eta (14)$, whereas the lower panels correspond to a release amplitude equal to 25% of the analytical threshold, $u_n=0.25\eta (14)$ (Color figure online)

Figure 6 presents the analogous comparison for a longer release interval, τ=14. Successful establishment of the infected population is observed for all three strains when using both the analytical release amplitude $u_n=\eta (14)$ and the reduced release amplitude $u_n=0.25\eta (14)$. As in the case τ=7, reducing the release effort primarily affects the transient dynamics rather than the long-term outcome.

The impact is again more pronounced for the wMelPop strain, which exhibits larger oscillations and a slower decline of the wild population before convergence to an infected-dominated regime. Nevertheless, successful establishment is observed for all strains under the reduced release amplitude.

These simulations show that the analytical threshold $\eta (\tau )$ is not sharp for the parameter regimes considered here. Although it guarantees establishment theoretically, successful invasion is still observed under release amplitudes equal to only 25% of the analytical prediction. The main consequence of reducing the release effort is therefore a slower replacement process, rather than a failure of establishment.

The numerical experiments performed during the release phase provide strong evidence that the analytical threshold $\eta (\tau )$ constitutes a conservative sufficient condition for *Wolbachia* establishment. Across all scenarios considered in this subsection, successful establishment was observed even when the release amplitude was reduced to 25% of the theoretical threshold. We next investigate whether these established populations remain persistent after the release program ends.

### Dynamics After the Releases Phase

**Fig. 7:**
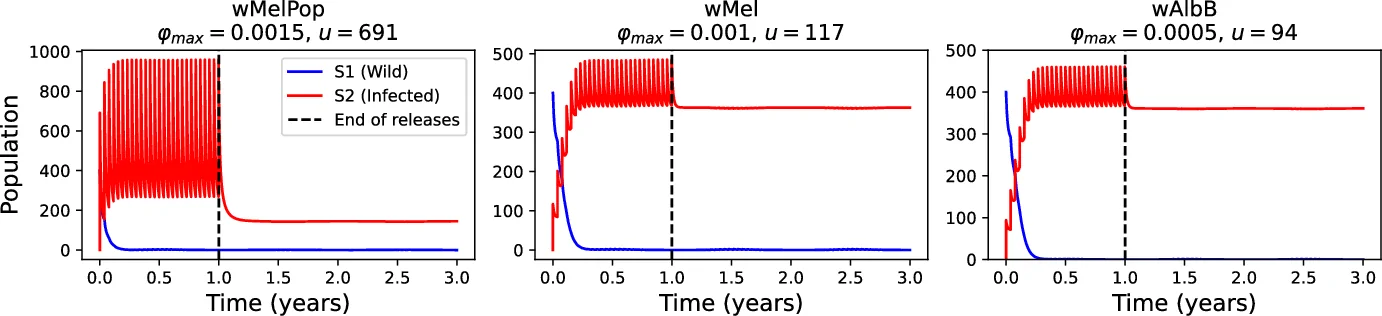
Population dynamics of wild and *Wolbachia*-infected mosquitoes under low temperature-induced infection loss after releases stop. The simulations consider τ=14 and $\varphi _{\max } = 0.0015$, 0.001, and 0.0005 for wMelPop, wMel, and wAlbB, respectively, with initial condition $(S_1(0),S_2(0))=(400,0)$. Releases occur only during the first year, and thereafter the system evolves under temperature-driven impulses alone. All three strains maintain a stable infected population and suppress the wild population throughout the three years (Color figure online)

**Fig. 8:**
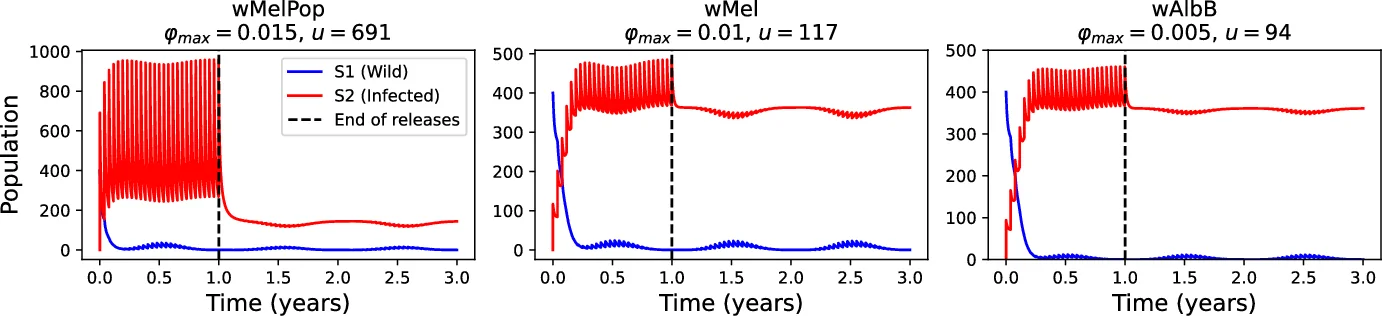
Population dynamics under moderate temperature-induced infection loss with τ=14 and $\varphi _{\max } = 0.015$, 0.01, and 0.005 for wMelPop, wMel, and wAlbB, respectively, with initial condition $(S_1(0),S_2(0))=(400,0)$. After the end of releases (year 1), the system exhibits stronger periodic fluctuations due to increased thermal decay of infection. Despite these oscillations, all three strains remain stable and continue to suppress the wild population over the three-year horizon (Color figure online)

**Fig. 9:**
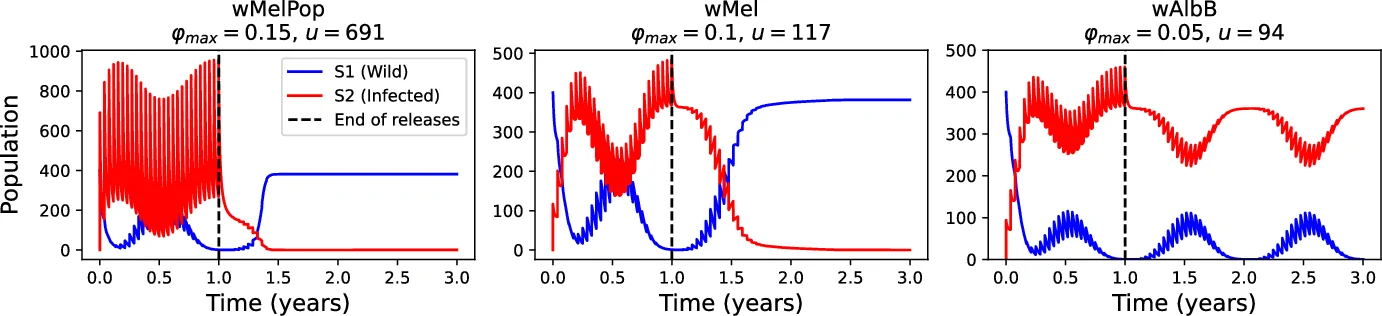
Population dynamics under high temperature-induced infection loss with τ=14 and $\varphi _{\max } = 0.15$, 0.1, and 0.05 for wMelPop, wMel, and wAlbB, respectively, with initial condition $(S_1(0),S_2(0))=(400,0)$. In this scenario, wMelPop and wMel fail to maintain *Wolbachia* infection after releases cease, leading to loss of the infected population and resurgence of the wild mosquitoes. In contrast, wAlbB remains stable despite stronger seasonal fluctuations, demonstrating its superior thermal tolerance (Color figure online)

In Figs. 7, 8, and 9, we examine the system’s behavior over three years, assuming that *Wolbachia*-infected females are released only during the first year. After this intervention phase, the system evolves without further releases, and the only remaining impulses are those associated with temperature-induced infection loss. We consider τ=14 and investigate three scenarios corresponding to low, moderate, and high temperature-induced infection loss.

#### Scenario 1: Low Infection Loss

Under low infection-loss intensities ($\varphi _{\max }=0.0015$, 0.001, and 0.0005 for wMelPop, wMel, and wAlbB, respectively), all three strains remain established after the release program ends (Fig. 7). The infected population converges to a stable positive equilibrium while the wild population remains suppressed, indicating that weak temperature-induced infection loss is insufficient to compromise long-term persistence.

#### Scenario 2: Moderate Infection Loss

Increasing the infection-loss intensity to $\varphi _{\max }=0.015$, 0.01, and 0.005 leads to more pronounced seasonal oscillations (Fig. 8). Nevertheless, all three strains remain established throughout the three-year period, indicating that moderate temperature-induced infection loss affects primarily the transient oscillations rather than long-term persistence.

#### Scenario 3: High Infection Loss

Under high infection-loss intensities ($\varphi _{\max }=0.15$, 0.1, and 0.05 for wMelPop, wMel, and wAlbB, respectively), pronounced differences emerge among the three strains (Fig. 9). For wMelPop and wMel, the high infection-loss rates prevent the infected population from remaining established after the release period, leading to a rapid decline of *Wolbachia* prevalence and a subsequent recovery of the wild population. In contrast, wAlbB remains substantially more resilient: although the infected population experiences stronger seasonal fluctuations, it still maintains a stable positive level throughout the post-release years. This outcome underscores the strong influence of thermal tolerance on long-term infection persistence once releases cease.

Across the three scenarios, the long-term persistence of *Wolbachia* after the end of the releases is strongly shaped by the magnitude of temperature-induced infection loss. Under low and moderate thermal stress (Scenarios 1 and 2), all three strains maintain a stable infected population and keep the wild population suppressed throughout the three-year period. However, when the loss rate becomes high (Scenario 3), the differences between strains become pronounced: wMelPop and wMel are unable to withstand the strong thermal pressure and rapidly lose their infected populations once releases stop, whereas wAlbB remains resilient and maintains infection persistence. These results highlight the combined importance of thermal tolerance and post-release dynamics, emphasizing that strain choice plays a decisive role in determining whether *Wolbachia* can remain established without continued intervention.

Our numerical findings are qualitatively consistent with experimental and field observations showing that elevated temperatures reduce *Wolbachia* density and weaken cytoplasmic incompatibility, particularly for heat-sensitive strains such as wMel (Ross et al. 2019; Gunasekaran et al. 2022; Ross et al. 2017). This biological behavior motivated the temperature-driven impulsive mechanism incorporated into our model. Consistent with these observations, our simulations predict long-term persistence for all strains under low and moderate thermal stress, whereas under high thermal stress only wAlbB remains established, reflecting its greater thermo tolerance.

### Robustness to Temperature and Imperfect Maternal Transmission

The analytical framework developed in Sects. 2 and 3 incorporates temperature effects exclusively through the infection-loss function φ(t) and assumes perfect maternal transmission of *Wolbachia*. Although these assumptions allow the analytical treatment of the model, experimental and field studies suggest that elevated temperatures may also affect mosquito demographic traits, while imperfect maternal transmission may occur under adverse environmental conditions.

To assess the robustness of our conclusions with respect to these assumptions, we consider two extensions of the model. First, the demographic parameters (birth and mortality rates) are allowed to vary seasonally with temperature. Second, maternal transmission is assumed to be imperfect, so that only a fraction of the offspring produced by infected females inherit the infection. These extensions considerably increase the complexity of the model and fall outside the analytical framework developed in Sect. 3. Therefore, no analytical results are derived for these modified systems, and their effects are investigated exclusively through numerical simulations.

To incorporate temperature effects into the demographic parameters, we introduce the normalized periodic function

$$ f(t)=\frac{\left( 1+\cos \left( \frac{2\pi (t-t_{\textrm{peak}})}{T_c}\right) \right) ^2}{4}, $$

which is obtained from the same seasonal signal used in the definition of the infection-loss function φ(t). Since $0\le f(t)\le 1$, the demographic parameters are modified according to

$$ \psi _i(t)=\psi _i\bigl (1-\varepsilon f(t)\bigr ), \qquad \delta _i(t)=\delta _i\bigl (1+\varepsilon f(t)\bigr ), $$

where $\varepsilon \in [0,1)$ measures the intensity of the temperature-dependent demographic variation. Thus, periods of stronger thermal stress simultaneously reduce birth rates and increase mortality rates. The impulsive mechanism associated with infection loss remains unchanged, so that seasonality affects the dynamics through both demographic variation and periodic infection loss.

To account for imperfect maternal transmission, we introduce a transmission efficiency parameter $\xi \in (0,1]$. Under this assumption, only a fraction ξ of the offspring produced by infected females inherit *Wolbachia*, whereas the remaining fraction 1-ξ is recruited into the uninfected population. Accordingly, the recruitment term associated with infected mosquitoes,

$$ \psi _2(t)S_2\left( 1-\frac{S_1+S_2}{K}\right) , $$

is partitioned as

$$ \xi \psi _2(t)S_2\left( 1-\frac{S_1+S_2}{K}\right) , $$

for infected offspring and

$$ (1-\xi )\psi _2(t)S_2\left( 1-\frac{S_1+S_2}{K}\right) , $$

for uninfected offspring.

**Fig. 10:**
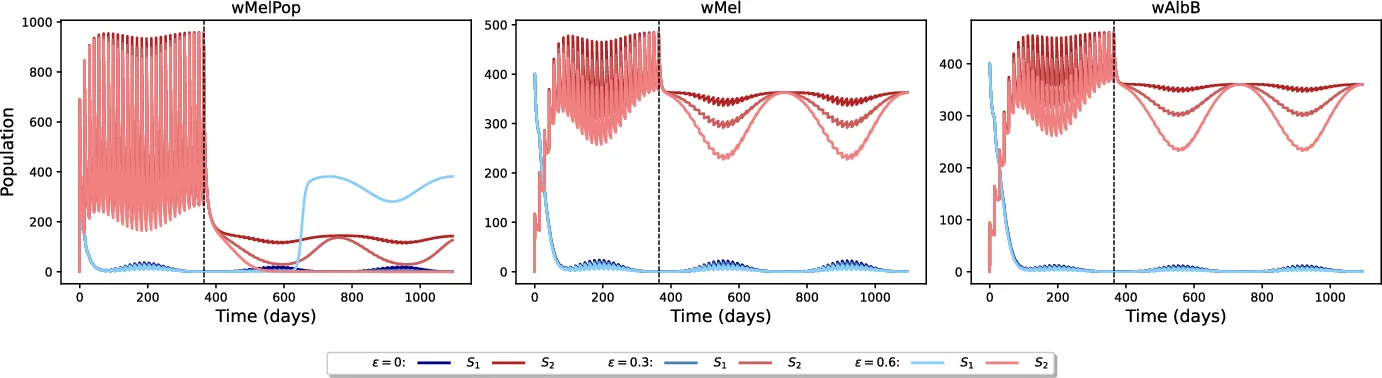
Population dynamics under temperature-dependent demographic variation for different values of ε with perfect maternal transmission (ξ=1). The initial condition is $(S_1(0),S_2(0))=(400,0)$. Periodic releases are performed every 14 days until day 365 using strain-specific release sizes of u=691 (wMelPop), u=117 (wMel), and u=94 (wAlbB) (Color figure online)

To assess the influence of temperature-dependent demographic variation, simulations are performed for different values of the sensitivity parameter ε, while maintaining perfect maternal transmission (ξ=1). The results are presented in Fig. 10.

Figure 10 shows that incorporating temperature-dependent demographic variation modifies the population dynamics, with more pronounced effects as ε increases. For wMel and wAlbB, although seasonal fluctuations become more evident during periods of higher thermal stress, the qualitative dynamics of the original model are preserved, and the infected population remains established after releases cease. In contrast, wMelPop exhibits a different outcome for ε=0.6: the infected population is unable to maintain establishment and eventually declines to zero, indicating a reversal of the replacement process. This behavior suggests that the higher demographic costs associated with wMelPop, when further amplified by temperature-dependent reductions in birth rates and increases in mortality rates, may compromise the persistence of the infected population under stronger demographic fluctuations.

While the previous simulations isolate the effects of temperature-dependent demographic variation under perfect maternal transmission, we next investigate the combined effects of temperature-dependent demographic changes and imperfect maternal transmission. This scenario provides a more comprehensive assessment of the persistence of *Wolbachia*-infected populations when both demographic performance and infection inheritance are affected.

**Fig. 11:**
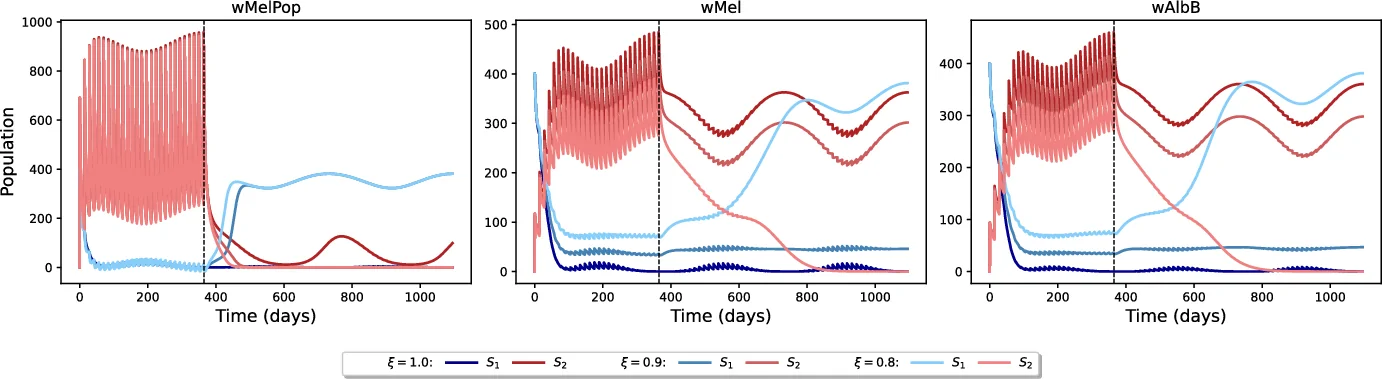
Population dynamics for different values of the maternal transmission efficiency ξ with ε=0.5. The initial condition is $(S_1(0),S_2(0))=(400,0)$. Periodic releases are performed every 14 days until day 365 using strain-specific release sizes of u=691 (wMelPop), u=117 (wMel), and u=94 (wAlbB) (Color figure online)

To examine these combined effects, the demographic sensitivity parameter was fixed at ε=0.5, while the maternal transmission efficiency ξ was varied. In addition to perfect transmission ξ=1.0, the values ξ=0.9 and ξ=0.8 were considered to explore the progressive loss of persistence as transmission efficiency decreases. In particular, ξ=0.8 was included to identify a regime in which persistence fails even for the strains that remain established at higher transmission efficiencies. The corresponding results are shown in Fig. 11.

For ξ=1.0, all three strains remain established after the release program despite the seasonal variation in demographic parameters. When the transmission efficiency is reduced to ξ=0.9, wMel and wAlbB remain established, whereas wMelPop is unable to maintain the infection, reflecting its higher demographic fitness costs. A more critical regime emerges for ξ=0.8, for which none of the three strains maintains long-term persistence: after releases cease, the infected populations progressively decline and the wild populations recover, indicating a reversal of the replacement process. Thus, the simultaneous action of temperature-dependent demographic changes and reduced maternal transmission can compromise population replacement even for wMel and wAlbB, which remain established under less severe conditions. These results emphasize that the combined effects of demographic and transmission-related mechanisms may produce qualitatively different establishment outcomes from those observed when each mechanism is considered under more favorable conditions.

Taken together, these numerical experiments show that temperature-dependent demographic variation can alter both seasonal population dynamics and, under stronger perturbations, the persistence of strains with higher fitness costs. More importantly, the combined action of demographic variation and imperfect maternal transmission can lead to a broader loss of infection persistence and reversal of the replacement process. These findings highlight the importance of considering the simultaneous effects of temperature on demographic performance and infection inheritance when assessing the long-term establishment of *Wolbachia*-infected populations.

## Final Remarks

This work combined analytical results and numerical simulations to investigate impulsive release strategies of *Aedes aegypti* mosquitoes infected with *Wolbachia* under temperature-dependent infection loss. The results show that long-term infection persistence emerges from the interaction between strain-specific biological characteristics, thermal stress, and operational release parameters.

The analytical results provide conditions for the existence and stability of periodic solutions and establish a release threshold associated with successful population replacement. The numerical simulations support these results and show that strain-specific fitness costs strongly influence the required release effort. In particular, wMelPop requires substantially larger releases than wMel and wAlbB, whereas wAlbB exhibits greater resilience under increasing thermal stress. Increasing the release interval and the intensity of temperature-induced infection loss further challenges long-term establishment, particularly after the release program ceases.

The model was deliberately formulated within a relatively simple framework that preserves analytical tractability while capturing the population-level mechanisms associated with impulsive releases and temperature-induced infection loss. This structure makes it possible to derive analytical conditions and thresholds that would be considerably more difficult to obtain in a more detailed formulation. However, some biologically relevant mechanisms, including temperature-dependent demographic variation and imperfect maternal transmission, are not explicitly incorporated into the analytical model. Their effects were therefore explored numerically.

These additional simulations reveal that relaxing the baseline assumptions can affect both population fluctuations and long-term establishment. Under perfect maternal transmission, temperature-dependent demographic variation primarily modifies the seasonal dynamics of wMel and wAlbB, while stronger demographic perturbations can compromise the persistence of wMelPop, which is associated with higher fitness costs. When demographic variation is combined with imperfect maternal transmission, more substantial changes in persistence emerge. In particular, reduced transmission efficiency can lead to the loss of infection even for wMel and wAlbB and to a reversal of the replacement process. Thus, the combined action of demographic and transmission-related effects may produce establishment outcomes that are not observed under the assumptions of the analytical framework.

These findings illustrate the complementary roles of analytical and numerical approaches in the present study. The analytical model provides a tractable framework for identifying mechanisms and deriving conditions governing *Wolbachia* establishment, whereas the extended numerical simulations allow the consequences of additional biological mechanisms to be examined without substantially increasing the complexity of the analytical formulation. Together, these approaches help identify both the conditions favoring successful population replacement and scenarios in which persistence may be compromised.

As with any mathematical model, the applicability of the results depends on the biological context and the quality of the available parameter estimates. The proposed framework should therefore be viewed as a tool for investigating release strategies and exploring potential intervention scenarios rather than as a source of precise predictions for every field setting. Future applications should be calibrated using local entomological and environmental data to ensure that the model adequately represents the population dynamics of the target region.

Future work may include extending the analytical framework to explicitly incorporate temperature-dependent demographic parameters and imperfect maternal transmission, as well as the incorporation of spatial heterogeneity, stochastic temperature variation, stage-structured mosquito populations, and optimal control approaches to further improve the design and evaluation of *Wolbachia*-based release strategies under realistic environmental conditions.

## Figures and Tables

**Fig. 12:**
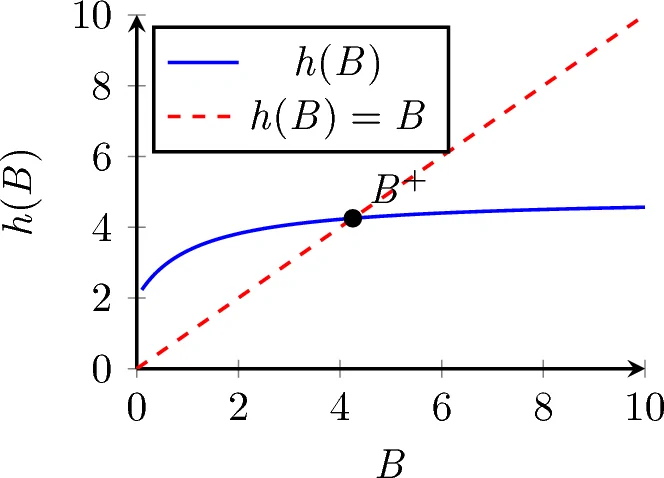
Illustration of the stroboscopic map *h*(*B*) and the identity line h(B)=B. The unique intersection point $B^+$ is globally attractive (Color figure online)

## Data Availability

All data generated or analyzed during this study are included in this published article.

## Appendix A Proof of Theorem 1

### Proof

To prove the theorem, we proceed in some steps.

(I) Local Lipschitz continuity.

Define

$$\begin{aligned} f_1(S_1,S_2)&= S_1\psi _1\left( 1 - \dfrac{S_1+S_2}{K}\right) \left( \dfrac{S_1+(1-\gamma )S_2}{S_1+S_2}\right) - \delta _1 S_1, \\ f_2(S_1,S_2)&= S_2\psi _2\left( 1 - \dfrac{S_1+S_2}{K}\right) - \delta _2 S_2. \end{aligned}$$

Let $f = (f_1,f_2)^\top$ and consider the domain

$$ D = \{(S_1,S_2) \in \mathbb {R}^2_{+}: S_1 + S_2 > 0\}. $$

In *D*, both $f_1$ and $f_2$ are rational functions formed by sums, products, and quotients of polynomials. Hence, they are $C^\infty$ on *D*.

The Jacobian matrix of *f* is given by

13 $$\begin{aligned} J(S_1,S_2) = \begin{bmatrix} \dfrac{\partial f_1}{\partial S_1} & \dfrac{\partial f_1}{\partial S_2} \\ \dfrac{\partial f_2}{\partial S_1} & \dfrac{\partial f_2}{\partial S_2} \end{bmatrix}, \end{aligned}$$

where

14 $$\begin{aligned} \frac{\partial f_1}{\partial S_1}&= \psi _1\left( 1 - \frac{S_1 + S_2}{K}\right) \left[ \left( \frac{S_1 + (1 - \gamma )S_2}{S_1 + S_2}\right) + \frac{\gamma S_1 S_2}{(S_1 + S_2)^2}\right] \nonumber \\&\qquad - \frac{\psi _1 S_1}{K}\left( \frac{S_1 + (1 - \gamma )S_2}{S_1 + S_2}\right) - \delta _1, \\\nonumber \frac{\partial f_1}{\partial S_2}&= S_1\psi _1\left[ -\frac{1}{K}\left( \frac{S_1 + (1 - \gamma )S_2}{S_1 + S_2}\right) - \left( 1 - \frac{S_1 + S_2}{K}\right) \frac{\gamma S_1}{(S_1 + S_2)^2}\right] , \\\nonumber \frac{\partial f_2}{\partial S_1}&= -\frac{\psi _2 S_2}{K}, \\\nonumber \frac{\partial f_2}{\partial S_2}&= \psi _2\left( 1 - \frac{S_1 + 2S_2}{K}\right) - \delta _2. \end{aligned}$$

All entries of $J(S_1,S_2)$ are continuous in *D*, so *f* is locally Lipschitz on every compact subset of *D*. Therefore, by the Picard–Lindelöf theorem, for any initial condition $(S_1(0), S_2(0)) \in D$, there exists a time $t_{\max } > 0$ and a unique solution $(S_1(t), S_2(t))$ defined on $[0, t_{\max })$.

(II) Positive invariance of $\mathbb {R}_+^2$.

If $S_1(0) = 0$ and $S_2(0) \ge 0$, then $f_1(0,S_2) = 0$, which implies $\frac{dS_1}{dt}(0) = 0$ and thus $S_1(t) \ge 0$ for t>0. Similarly, if $S_2(0) = 0$ and $S_1(0) \ge 0$, then $f_2(S_1,0) = 0$, implying $\frac{dS_2}{dt}(0) = 0$. Hence, trajectories starting in $\mathbb {R}_+^2$ remain there, since the flow is tangent or directed inward along the coordinate axes. Therefore, the nonnegative orthant $\mathbb {R}_+^2$ is positively invariant.

(III) Boundedness of solutions.

Let $$S(t) = S_1(t) + S_2(t)$$. Then,

$$\begin{aligned} \frac{dS}{dt}&= f_1 + f_2 \\&= S_1\psi _1\left( 1 - \frac{S}{K}\right) \left( \frac{S_1 + (1-\gamma )S_2}{S_1 + S_2}\right) + S_2\psi _2\left( 1 - \frac{S}{K}\right) - \delta _1 S_1 - \delta _2 S_2. \end{aligned}$$

Since $0 \le \frac{S_1 + (1-\gamma )S_2}{S_1 + S_2} \le 1$, we obtain

$$ \frac{dS}{dt} \le \psi S\left( 1 - \frac{S}{K}\right) - \delta S, $$

where $$\psi = \max \{\psi _1, \psi _2\}$$ and $$\delta = \min \{\delta _1, \delta _2\}$$.

To analyze this inequality, consider the comparison system

$$\begin{aligned} \frac{dy}{dt}&= \psi y\left( 1 - \frac{y}{K}\right) - \delta y,\\ y(0)&= S(0). \end{aligned}$$

This equation can be written as

$$ \frac{dy}{dt} = y(r - a y), \quad \text {where } r = \psi - \delta , \quad a = \frac{\psi }{K}. $$

This is a logistic equation with an analytical solution

$$ y(t) = \frac{y(0)r e^{rt}}{a y(0)(e^{rt} - 1) + r}, $$

which satisfies $0< y(t) < K\left( 1 - \frac{\delta }{\psi }\right) {=}{:} y^*$ for all t>0 provided $\psi > \delta$. By the Comparison Lemma, S(t)≤y(t) for all *t* in the domain of existence. Hence, *S*(*t*) is uniformly bounded by $\max \{S(0), y^*, K\}$. Finally, since *S*(*t*) remains uniformly bounded for all t≥0, the solution $(S_1(t),S_2(t))$ stays in a compact subset of $\mathbb {R}^2_+$. Because the vector field is locally Lipschitz, no finite–time blow-up can occur, and the solution can be extended indefinitely. Therefore, $(S_1(t), S_2(t))$ exists for all t≥0.

(IV) Solution for all t≥0.

From the above results, for any nonnegative initial condition, there exists a unique global solution $(S_1(t),S_2(t)) \in \mathbb {R}^2_+$ for all t≥0, and both components remain bounded. □

### Remark 1

Behavior at the origin (0, 0).

The function

$$ g(S_1,S_2) = \frac{S_1 + (1-\gamma )S_2}{S_1 + S_2} $$

is undefined at (0, 0). However, its limits along the coordinate axes are

$$ \lim _{S_2 \rightarrow 0^+} g(S_1,S_2) = 1, \qquad \lim _{S_1 \rightarrow 0^+} g(S_1,S_2) = 1 - \gamma . $$

Thus, *g* admits a continuous extension to (0, 0) by defining, for example,

g(0,0)=1,

or any value in the interval $[1-\gamma ,\,1]$. With this extension, *f* becomes continuous on $\mathbb {R}^2_+$, and the trivial solution $(S_1,S_2) \equiv (0,0)$ is an equilibrium point. The uniqueness and positivity of nontrivial solutions are not affected by this extension.

## Appendix B Proof of Theorem 2

### Proof

The equilibrium points of system (1) are obtained by setting

$$ \dfrac{dS_1}{dt} = \dfrac{dS_2}{dt} = 0, $$

which yields the following system of algebraic equations:

15 $$\begin{aligned} S_1\psi _1\left( 1 - \dfrac{1}{K}(S_1 + S_2) \right) \left( \dfrac{S_1+(1-\gamma )S_2}{S_1+S_2} \right) - \delta _1 S_1&= 0, \end{aligned}$$

16 $$\begin{aligned} S_2\psi _2 \left( 1 - \dfrac{1}{K}(S_1 + S_2) \right) - \delta _2 S_2&= 0. \end{aligned}$$

From Eq. (16), it follows that either $S_2 = 0$ or

$$ \psi _2 \left( 1 - \dfrac{1}{K}(S_1 + S_2) \right) = \delta _2. $$

In the latter case, we obtain

17 $$\begin{aligned} S^* = S_1 + S_2 = K\left( 1 - \dfrac{\delta _2}{\psi _2} \right) , \end{aligned}$$

which allows us to express $S_2$ as a function of $S_1$:

$$ S_2 = K\left( 1 - \dfrac{\delta _2}{\psi _2} \right) - S_1. $$

By substituting this expression into Eq. (15) and solving for $S_1$, while introducing $R_1 = \frac{\psi _1}{\delta _1}$ and $R_2 = \frac{\psi _2}{\delta _2}$, assumed to be greater than 1 under conditions (2)-(3), we obtain

$$ \hat{S}_1 = \left( 1 - \frac{1}{R_2} \right) \frac{K}{\gamma } \left( \frac{R_2}{R_1} - (1 - \gamma ) \right) , $$

from which we obtain

$$ \hat{S}_2 = K\left( 1 - \dfrac{1}{R_2} \right) - \hat{S}_1, $$

or equivalently,

$$ \hat{S}_2 = \left( 1 - \frac{1}{R_2} \right) K\left[ 1 - \frac{1}{\gamma } \left( \frac{R_2}{R_1} - (1 - \gamma ) \right) \right] . $$

Therefore, the coexistence equilibrium point is given by $E_3 = (\hat{S}_1, \hat{S}_2)$. For this point to exist, both coordinates must be positive. Hence, $\hat{S}_1 > 0$ if and only if $\frac{R_2}{R_1} > (1 - \gamma )$, and $\hat{S}_2 > 0$ if and only if $\frac{R_2}{R_1} < 1$. Consequently, the coexistence equilibrium $E_3$ exists if and only if

$$ 1 - \gamma< \frac{R_2}{R_1} < 1. $$

Next, we analyze the cases in which one of the populations goes extinct:

- If $S_2 = 0$, Eq. (15) reduces to
  $$ S_1\psi _1 \left( 1 - \dfrac{S_1}{K} \right) - \delta _1 S_1 = 0, $$
  which yields $S_1^* = 0$ or $S_1^* = K\left( 1 - \dfrac{1}{R_1} \right)$, provided that $\psi _1 > \delta _1$.
- If $S_1 = 0$, from Eq. (17) we obtain
  $$ S_2^* = K\left( 1 - \dfrac{1}{R_2} \right) , $$
  with $$\psi _2 > \delta _2$$.

Therefore, the three equilibrium points of the system can be explicitly written in terms of the model parameters as follows:

- $$E_1 = \left( K\left( 1 - \dfrac{1}{R_1} \right) , 0\right) $$,
- $$E_2 = \left( 0, K\left( 1 - \dfrac{1}{R_2} \right) \right) $$,
- $$E_3 = (\hat{S}_1, \hat{S}_2)$$, where
  $$\begin{aligned} \hat{S}_1&= \left( 1 - \frac{1}{R_2} \right) \frac{K}{\gamma } \left( \frac{R_2}{R_1} - (1 - \gamma ) \right) , \\ \hat{S}_2&= \left( 1 - \frac{1}{R_2} \right) K\left[ 1 - \frac{1}{\gamma } \left( \frac{R_2}{R_1} - (1 - \gamma ) \right) \right] . \end{aligned}$$

We now proceed to analyze the local stability of the three equilibrium points obtained. For this purpose, we consider the Jacobian matrix of system (1), which is given by (13)–(14).

- For the *Wolbachia*-free equilibrium $E_1 = \left( K\left( 1 - \dfrac{1}{R_1} \right) , 0\right)$, we have:
  $$ J(E_1) = \begin{bmatrix} \delta _1 - \psi _1 & \delta _1(2-\gamma ) - \psi _1 \\ 0 & \dfrac{\psi _2}{R_1} - \delta _2 \end{bmatrix}. $$
  The eigenvalues of the characteristic polynomial $p(\lambda ) = \det (J(E_1) - \lambda I)$ are given by $\lambda _1 = \delta _1 - \psi _1$ and $\lambda _2 = \dfrac{\psi _2}{R_1} - \delta _2$. Note that $\lambda _1 < 0$ due to the condition (2) imposed on the model. Therefore, for the equilibrium $E_1$ to be locally asymptotically stable, it is necessary that $\lambda _2 < 0$, which leads to the condition
  $$ R_1 > R_2. $$
- For the wild-type-free equilibrium $E_2 = \left( 0, K\left( 1 - \dfrac{1}{R_2} \right) \right)$, we obtain:
  $$ J(E_2) = \begin{bmatrix} \psi _1(1-\gamma )\dfrac{1}{R_2} - \delta _1 & 0 \\ \delta _2 - \psi _2 & \delta _2 - \psi _2 \end{bmatrix}. $$
  In this case, the eigenvalues of the characteristic polynomial $p(\lambda ) = \det (J(E_2) - \lambda I)$ are $\lambda _1 = \psi _1(1-\gamma )\dfrac{1}{R_2} - \delta _1$ and $\lambda _2 = \delta _2 - \psi _2$. As in the previous case, $\lambda _2 < 0$ by condition (2). Hence, $E_2$ will be locally asymptotically stable if $\lambda _1 < 0$, that is, if
  $$ R_2 > (1 - \gamma )R_1. $$
- To analyze the local stability of the coexistence equilibrium, we consider the trace and determinant of the Jacobian matrix
  $$ J(E_3) = \begin{bmatrix} \displaystyle \left. \frac{\partial f_1}{\partial S_1}\right| _{E_3} & \displaystyle \left. \frac{\partial f_1}{\partial S_2}\right| _{E_3} \\ \displaystyle \left. \frac{\partial f_2}{\partial S_1}\right| _{E_3} & \displaystyle \left. \frac{\partial f_2}{\partial S_2}\right| _{E_3} \end{bmatrix}. $$
  Let the equilibrium point be $E_3 = (\hat{S}_1, \hat{S}_2)$, where
  $$\begin{aligned} \hat{S}_1&= \left( 1 - \frac{1}{R_2} \right) \frac{K}{\gamma } \left( \frac{R_2}{R_1} - (1 - \gamma ) \right) , \\ \hat{S}_2&= \left( 1 - \frac{1}{R_2} \right) K\left[ 1 - \frac{1}{\gamma } \left( \frac{R_2}{R_1} - (1 - \gamma ) \right) \right] . \end{aligned}$$
  Then, the entries of the Jacobian matrix evaluated at $E_3$ are given by
  $$\begin{aligned} \left. \frac{\partial f_1}{\partial S_1}\right| _{E_3}&= \psi _1\left( 1 + \frac{c + \gamma - 1}{\gamma }\right) - \frac{\psi _1}{\gamma }\left( 1 - \frac{1}{R_2}\right) (c + \gamma - 1)(2 - c) - \delta _1, \\ \left. \frac{\partial f_1}{\partial S_2}\right| _{E_3}&= -\frac{\delta _2}{\psi _2}\frac{(c + \gamma - 1)^2}{\gamma }\psi _1 - \left( 1 - \frac{1}{R_2}\right) \frac{(c + \gamma - 1)\psi _1}{\gamma },\\ \left. \frac{\partial f_2}{\partial S_1}\right| _{E_3}&= -\frac{(\psi _2 - \delta _2)(1 - c)}{\gamma }, \\ \left. \frac{\partial f_2}{\partial S_2}\right| _{E_3}&= (\psi _2 - \delta _2) - \frac{K}{\gamma }(\psi _2 - \delta _2)(1 + \gamma - c), \end{aligned}$$
  where $c = \dfrac{R_2}{R_1}$. Hence, the determinant of the Jacobian matrix at $E_3$ is
  $$ \det (J(E_3)) = \frac{(\psi _2-\delta _2) (\delta _1 \psi _2 - \delta _2 \psi _1) (\delta _1 \psi _2 + \delta _2 \gamma \psi _1 - \delta _2 \psi _1)}{\delta _2\gamma \psi _1 \psi _2}. $$
  For the equilibrium $E_3$ to be locally asymptotically stable, the following conditions must hold:
  $$ \operatorname {tr}(J(E_3)) < 0 \quad \text {and} \quad \det (J(E_3)) > 0. $$
  However, the existence of $E_3$ requires that
  $$ 1 - \gamma< \dfrac{R_2}{R_1}< 1. $$
  Since $\psi _2 > \delta _2$, we have
  $$\begin{aligned} \delta _1 \psi _2 - \delta _2 \psi _1&= \psi _1 \delta _2 (c - 1),\\ \delta _1\psi _2+\delta _2\gamma \psi _1 - \delta _2 \psi _1&= \psi _1 \delta _2 (c + \gamma - 1). \end{aligned}$$
  We can rewrite,
  $$ \det (J(E_3)) = \frac{(\psi _2-\delta _2)(c-1)(c+\gamma -1)\psi _1\delta _2}{\gamma \psi _2} < 0, $$
  since c-1<0 by the existence condition of the equilibrium point. Consequently, if the coexistence equilibrium $E_3$ exists, then $\det (J(E_3)) < 0$, and thus $E_3$ is a saddle point (unstable).

□

## Appendix C Proof of Theorem 5

### Proof

Let,

$$\begin{aligned} B(t) = \dfrac{c \bar{K} e^{r_2 t}}{ce^{r_2 t}-1}, \end{aligned}$$

be the solution of

$$\begin{aligned} \dfrac{dB}{dt}(t) = B\psi _2\left( 1 - \dfrac{B}{K}\right) - \delta _2 B \quad t \ne (n + l)\tau , \text  t \ne (n + 1)\tau , \end{aligned}$$

the first equation of (7), where $r_2 = \psi _2-\delta _2$, $\bar{K} = \frac{Kr_2}{\psi _2}$ and $c \in \mathbb {R}$ is a constant determined by the initial conditions of the problem. For t=nτ and t=(n+l)τ, we denote the initial values at these times as $B(n\tau ^+)$ and $B((n+l)\tau ^+)$, respectively. The constant *c* is then given by:

$$\begin{aligned} c = {\left\{ \begin{array}{ll} \dfrac{B(n\tau ^+) e^{-r_2 n\tau }}{B(n\tau ^+) - \bar{K}}, & \text {for } t = n\tau , \\ \dfrac{B((n + l)\tau ^+) e^{-r_2 (n + l)\tau }}{B((n + l)\tau ^+) - \bar{K}}, & \text {for } t = (n + l)\tau . \end{array}\right. } \end{aligned}$$

then,

18 $$\begin{aligned} B(t) = {\left\{ \begin{array}{ll} \dfrac{\bar{K} B(n\tau ^+) e^{r_2 (t-n\tau )}}{B(n\tau ^+)\left( e^{r_2 (t-n\tau )} -1\right) +\bar{K}}, \quad n\tau< t \le (n+l)\tau ,\\ \dfrac{\bar{K} B((n+l)\tau ^+) e^{r_2 (t-(n+l)\tau )}}{B((n+l)\tau ^+)\left( e^{r_2 (t-(n+l)\tau )} -1\right) +\bar{K}}, \quad (n+l)\tau < t \le (n+1)\tau , \end{array}\right. } \end{aligned}$$

is the solution of system (6) between the pulses. At the pulse moments, when t=(n+l)τ with n≥0, the second equation of system (6) yields the following difference equation:

19 $$\begin{aligned} B((n+l)\tau ^+) =&(1-\varphi )B((n+l)\tau ^-)\nonumber \\ =&(1-\varphi )\dfrac{\bar{K} B(n\tau ^+) e^{r_2 ((n+l)\tau -n\tau )}}{B(n\tau ^+)\left( e^{r_2 ((n+l)\tau -n\tau )} -1\right) +\bar{K}}\nonumber \\ =&(1-\varphi )\dfrac{\bar{K} B(n\tau ^+) e^{r_2 l \tau }}{B(n\tau ^+)\left( e^{r_2 l\tau } -1\right) +\bar{K}}. \end{aligned}$$

Similarly, at the next pulse time t=(n+1)τ, n≥0, the third equation of system (6) gives the difference equation:

20 $$\begin{aligned} B((n+1)\tau ^+) =&B((n+1)\tau ^-) + u_n\nonumber \\ =&\dfrac{\bar{K} B((n+l)\tau ^+) e^{r_2 ((n+1)\tau -(n+l)\tau )}}{B((n+l)\tau ^+)\left( e^{r_2 ((n+1)\tau -(n+l)\tau )} -1\right) +\bar{K}} + u_n\nonumber \\ =&\dfrac{\bar{K} B((n+l)\tau ^+) e^{r_2(1-l)\tau }}{B((n+l)\tau ^+)\left( e^{r_2(1-l)\tau } -1\right) +\bar{K}} + u_n. \end{aligned}$$

Substituting Eq. (19) into (20), we obtain the recursive relation between consecutive pulse states:

$$\begin{aligned} B((n+1)\tau ^+) =&\dfrac{\bar{K} \left( (1-\varphi )\dfrac{\bar{K} B(n\tau ^+) e^{r_2 l \tau }}{B(n\tau ^+)\left( e^{r_2 l\tau } -1\right) +\bar{K}}\right) e^{r_2(1-l)\tau }}{\left( (1-\varphi )\dfrac{\bar{K} B(n\tau ^+) e^{r_2 l \tau )}}{B(n\tau ^+)\left( e^{r_2 l\tau )} -1\right) +\bar{K}}\right) \left( e^{r_2(1-l)\tau } -1\right) +\bar{K}} + u_n,\\ =&\frac{\bar{K}e^{r_2\tau }(1-\varphi )B(n\tau ^+)}{B(n\tau ^+)\left( (1-\varphi )e^{r_2\tau } + \varphi e^{r_2l\tau }-1\right) +\bar{K}} +u_n. \end{aligned}$$

We can rewrite it as

21 $$\begin{aligned} B^{n+1} = h(B^n) := \frac{\bar{K}e^{r_2\tau }(1-\varphi )B^n}{B^n\left( (1-\varphi )e^{r_2\tau } + \varphi e^{r_2l\tau }-1\right) +\bar{K}} + u_n, \end{aligned}$$

with $B^n:= B(n\tau ^+)$, which is the stroboscopic map of (6). Set,

$$\begin{aligned} h(B) = \frac{\bar{K}e^{r_2\tau }(1-\varphi )B}{B\left( (1-\varphi )e^{r_2\tau } + \varphi e^{r_2l\tau }-1\right) +\bar{K}} +u_n, \end{aligned}$$

Note that equation (21) has a single positive fixed point, since the equilibrium condition

$$\begin{aligned} h(B) = B, \end{aligned}$$

which determines the τ-periodic fixed point of the recursive Eq. (21), yields a unique positive solution through algebraic manipulation. Then,

22 $$\begin{aligned} A B^2 - \left( \bar{K}\left( (1-\varphi )e^{r_2\tau } -1\right) + u_nA\right) B - u_n\bar{K} = 0, \end{aligned}$$

where

23 $$\begin{aligned} A := (1-\varphi )e^{r_2\tau } + \varphi e^{r_2 l \tau } - 1, \end{aligned}$$

and the discriminant Δ is

$$\begin{aligned} \Delta := \big (\bar{K}\big ((1-\varphi )e^{r_2\tau } - 1\big ) + u_n A\big )^2 + 4 u_n \bar{K} A. \end{aligned}$$

Since A>0 and Δ>0, the unique positive solution of (22) is

24 $$\begin{aligned} B^{+} = \dfrac{\bar{K}\big ((1-\varphi )e^{r_2\tau } - 1\big ) + u_n A + \sqrt{\big (\bar{K}\big ((1-\varphi )e^{r_2\tau } - 1\big ) + u_n A\big )^2 + 4 u_n \bar{K} A}}{2A}. \end{aligned}$$

Now, substituting $B^+$ into the first equation of (18), we obtain

$$\begin{aligned} \bar{B}(t)&= \dfrac{\bar{K} B^+ e^{r_2 (t-n\tau )}}{B^+\left( e^{r_2 (t-n\tau )} -1\right) +\bar{K}}, \quad n\tau < t \le (n+l)\tau , \end{aligned}$$

and for $(n + l)\tau < t \le (n + 1)\tau$, we have

$$\begin{aligned} \bar{B}(t)&= \dfrac{\bar{K} B^* e^{r_2 (t-(n+l)\tau )}}{B^*\left( e^{r_2 (t-(n+l)\tau )} -1\right) +\bar{K}}, \end{aligned}$$

where $B^* = \dfrac{(1-\varphi )\bar{K}B^+e^{r_2l\tau }}{B^+\left( e^{r_2l\tau }-1\right) +\bar{K}}.$ Thus, the function

$$\begin{aligned} \bar{B}(t) = {\left\{ \begin{array}{ll} \dfrac{\bar{K} B^+e^{r_2 (t-n\tau )}}{B^+\left( e^{r_2 (t-n\tau )} -1\right) +\bar{K}}, \quad n\tau< t \le (n+l)\tau ,\\ \dfrac{\bar{K} B^* e^{r_2 (t-(n+l)\tau )}}{B^*\left( e^{r_2 (t-(n+l)\tau )} -1\right) +\bar{K}}, \quad (n+l)\tau < t \le (n+1)\tau , \end{array}\right. } \end{aligned}$$

defines the unique positive τ-periodic solution of system (6).

Furthermore, the function *h*(*B*) is strictly increasing for B>0, since it is composed of rational expressions with positive coefficients. Moreover,

$$\begin{aligned} \lim _{B \rightarrow 0^+} h(B) = u_n > 0 \quad \text {and} \quad \lim _{B \rightarrow \infty } h(B) = \dfrac{\bar{K}e^{r_2\tau }(1-\varphi )}{(1-\varphi )e^{r_2\tau } + \varphi e^{r_2l\tau } - 1} + u_n. \end{aligned}$$

Hence, *h*(*B*) intersects the identity line h(B)=B only once at $B = B^+$, and we conclude that:

$$\begin{aligned} h(B)< B&\quad \text {for all } B> B^+, \\ h(B) > B&\quad \text {for all } B < B^+. \end{aligned}$$

This implies that the sequence $\{B^n\}$ defined recursively by (21) converges monotonically to $B^+$ from any initial condition $B^0 > 0$. Thus, $B^+$ is globally asymptotically stable (Fig. 12).

Since $B^+$ is the unique globally attractive fixed point of the stroboscopic map, and the solution $\bar{B}(t)$ is constructed from it, we conclude that any solution of (6) with $B(0^+) > 0$ converges to $\bar{B}(t)$ as $t \rightarrow \infty$. □

## Footnotes

1 The eigenvalues of the monodromy matrix associated with a periodic orbit determine the behavior of infinitesimal perturbations over one period and are called the *Floquet multipliers* of the periodic orbit.

## Code Availability

The computational codes used to perform the numerical simulations are available from the corresponding author upon reasonable request.

## References

[CR1] Ahebwa A, Hii J, Neoh K-B, Chareonviriyaphap T (2023) Aedes aegypti and aedes albopictus (Diptera: Culicidae) ecology, biology, behaviour, and implications on arbovirus transmission in Thailand. One Health 16:100555. 10.1016/j.onehlt.2023.100555 PMC10288100 37363263

[CR2] Almeida L, Duprez M, Privat Y, Vauchelet N (2019) Mosquito population control strategies for fighting against arboviruses. Math Biosci Eng 16(6):6274–6297. 10.3934/mbe.2019313 31698562

[CR3] Almeida L, Privat Y, Strugarek M, Vauchelet N (2019) Optimal releases for population replacement strategies: application to Wolbachia. SIAM J Math Anal 51(4):3170–3194. 10.1137/18M1189841

[CR4] Almeida L, Duprez M, Privat Y, Vauchelet N (2022) Optimal control strategies for the sterile mosquitoes technique. J Differential Equations 311:229–266. 10.1016/j.jde.2021.12.002

[CR5] Almeida L, Bellver-Arnau J, Privat Y, Rebelo C (2024) Vector-borne disease outbreak control via instant releases. J Math Biol 89(6):63. 10.1007/s00285-024-02159-9 PMC11557670 39532731

[CR6] Alves JC, Oliva SM, Schaerer CE (2026) Optimizing impulsive releases: a species competition model. Appl Math Model 151:116517. 10.1016/j.apm.2025.116517

[CR7] Ant TH, Herd CS, Geoghegan V, Hoffmann AA, Sinkins SP (2018) The wolbachia strain w au provides highly efficient virus transmission blocking in aedes aegypti. PLoS Pathog 14(1):1006815. 10.1371/journal.ppat.1006815 PMC5784998 29370307

[CR8] Axford JK, Ross PA, Yeap HL, Callahan AG, Hoffmann AA (2016) Fitness of walbb wolbachia infection in aedes aegypti: parameter estimates in an outcrossed background and potential for population invasion. Am J Trop Med Hyg 94(3):507. 10.4269/ajtmh.15-0608 PMC4775882 26711515

[CR9] Bainov D, Simeonov P (1993) Impulsive differential equations: periodic solutions and applications. Pitman monographs and surveys in pure and applied mathematics, vol 66. Longman Scientific & Technical, Harlow

[CR10] Blackwood JC, Vargas R Jr, Fauvergue X (2018) A cascade of destabilizations: combining Wolbachia and Allee effects to eradicate insect pests. J Anim Ecol 87(1):59–72. 10.1111/1365-2656.12756 28913925

[CR11] Bliman P-A, Cardona-Salgado D, Dumont Y, Vasilieva O (2019) Implementation of control strategies for sterile insect techniques. Math Biosci 314:43–60. 10.1016/j.mbs.2019.06.002 31176705

[CR12] Campo-Duarte DE, Vasilieva O, Cardona-Salgado D (2017) Optimal control for enhancement of Wolbachia frequency among aedes aegypti females. Int J Pure Appl Math 112(2):219–238. 10.12732/ijpam.v112i2.1

[CR13] Campo-Duarte DE, Vasilieva O, Cardona-Salgado D, Svinin M (2018) Optimal control approach for establishing wmelpop wolbachia infection among wild aedes aegypti populations. J Math Biol 76:1907–1950. 10.1007/s00285-018-1213-2 29429122

[CR14] Cardona-Salgado D, Campo-Duarte DE, Sepulveda-Salcedo LS, Vasilieva O (2020) Wolbachia-based biocontrol for dengue reduction using dynamic optimization approach. Appl Math Model 82:125–149. 10.1016/j.apm.2020.01.032

[CR15] Carson J, Erriah B, Herodotou S, Shtukenberg AG, Smith L, Ryazanskaya S, Ward MD, Kahr B, Lees RS (2023) Overcoming insecticide resistance in anopheles mosquitoes by using faster-acting solid forms of deltamethrin. Malar J 22(1):129. 10.1186/s12936-023-04554-x PMC10120210 37081532

[CR16] Cheong YL, Ghazali SM, Hashim MHM, Ibrahim MKC, Amran A, Tiunh TY, Lim HL, Cheah YK, Gill BS, Lim KH (2025) Exploring 97 years of aedes aegypti as the vector for dengue, yellow fever, zika, and chikungunya (Diptera: Culicidae): Scientometric analysis. Interact J Med Res 14(1):65844. 10.2196/65844 PMC12059494 40267478

[CR17] Dianavinnarasi J, Raja R, Alzabut J, Niezabitowski M, Bagdasar O (2021) Controlling Wolbachia transmission and invasion dynamics among aedes aegypti population via impulsive control strategy. Symmetry 13(3):434. 10.3390/sym13030434

[CR18] Dorigatti I, McCormack C, Nedjati-Gilani G, Ferguson NM (2018) Using Wolbachia for dengue control: insights from modelling. Trends Parasitol 34(2):102–113. 10.1016/j.pt.2017.11.002 PMC5807169 29183717

[CR19] Dos Santos GR, Durovni B, Saraceni V, Riback TI, Pinto SB, Anders KL, Moreira LA, Salje H (2022) Estimating the effect of the wmel release programme on the incidence of dengue and chikungunya in rio de janeiro, brazil: a spatiotemporal modelling study. Lancet Infect Dis 22(11):1587–1595. 10.1016/S1473-3099(22)00436-4 PMC9630156 36182679

[CR20] Gesto JSM, Pinto SB, Dias FBS, Peixoto J, Costa G, Kutcher S, Montgomery J, Green BR, Anders KL, Ryan PA, Simmons CP, O’Neill SL, Moreira LA (2021) Large-scale deployment and establishment of wolbachia into the aedes aegypti population in Rio de Janeiro, Brazil. Front Microbiol 12:711107. 10.3389/fmicb.2021.711107 PMC8356046 34394061

[CR21] Gonzales JLO, Santos Benedito A, Oliveira Florentino H, Ferreira CP, Cardona-Salgado D, Sepulveda-Salcedo LS, Vasilieva O (2025) Optimization approaches to wolbachia-based biocontrol. Appl Math Model 137:115663. 10.1016/j.apm.2024.115663

[CR22] Gunasekaran K, Sadanandane C, Panneer D, Kumar A, Rahi M, Dinesh S, Vijayakumar B, Krishnaraja M, Subbarao SK, Jambulingam P (2022) Sensitivity of w mel and w albb wolbachia infections in aedes aegypti Puducherry (Indian) strains to heat stress during larval development. Parasit Vectors 15(1):221. 10.1186/s13071-022-05345-0 PMC9215077 35729601

[CR23] Huang M, Song X, Li J (2017) Modelling and analysis of impulsive releases of sterile mosquitoes. J Biol Dyn 11(1):147–171. 10.1080/17513758.2016.1254286 27852161

[CR24] Huang M, You L, Liu S, Song X (2021) Impulsive release strategies of sterile mosquitos for optimal control of wild population. J Biol Dyn 15(1):151–176. 10.1080/17513758.2021.1887380 33600289

[CR25] Kaur R, Meier CJ, McGraw EA, Hillyer JF, Bordenstein SR (2024) The mechanism of cytoplasmic incompatibility is conserved in wolbachia-infected aedes aegypti mosquitoes deployed for arbovirus control. PLoS Biol 22(3):3002573. 10.1371/journal.pbio.3002573 PMC11014437 38547237

[CR26] Lakshmikantham V, Bainov DD, Simeonov PS (1989) Theory of impulsive differential equations. Series in modern applied mathematics, vol 6. World Scientific, Singapore (1989). 10.1142/0906

[CR27] Lees RS, Fornadel C, Snetselaar J, Wagman J, Spiers A (2023) Insecticides for mosquito control: improving and validating methods to strengthen the evidence base. MDPI. 10.3390/insects14020116 PMC9961412 36835685

[CR28] Li J, Ai S (2020) Impulsive releases of sterile mosquitoes and interactive dynamics with time delay. J Biol Dyn 14(1):289–307. 10.1080/17513758.2020.1748239 32301687

[CR29] Li Y, Zhao H, Cheng Y (2024) Modeling mosquito control by an impulsive reaction-diffusion mosquito model with periodic evolution domain. Commun Nonlinear Sci Numer Simul 130:107738. 10.1016/j.cnsns.2023.107738

[CR30] Lim A, Shearer FM, Sewalk K, Pigott DM, Clarke J, Ghouse A, Judge C, Kang H, Messina JP, Kraemer MU et al (2025) The overlapping global distribution of dengue, chikungunya, zika and yellow fever. Nat Commun 16(1):3418. 10.1038/s41467-025-58609-5 PMC11986131 40210848

[CR31] Liu Z, Chen T, Zhou T (2023) Analysis of impulse release of Wolbachia to control nilaparvata lugens. Commun Nonlinear Sci Numer Simul 116:106842. 10.1016/j.cnsns.2022.106842

[CR32] Lopes LE, Ferreira CP, Oliva SM (2023) Exploring the impact of temperature on the efficacy of replacing a wild aedes aegypti population by a Wolbachia-carrying one. Appl Math Model 123:392–405

[CR33] Maciel-de-Freitas R, Sauer FG, Kliemke K, Garcia GA, Pavan MG, David MR, Schmidt-Chanasit J, Hoffmann A, Lühken R (2024) Wolbachia strains w mel and w albb differentially affect aedes aegypti traits related to fecundity. Microbiol Spectrum 12(4):00128–24. 10.1128/spectrum.00128-24 PMC10986601 38483475

[CR34] Mancini MV, Ant TH, Herd CS, Martinez J, Murdochy SM, Gingell DD, Mararo E, Johnson PC, Sinkins SP (2021) High temperature cycles result in maternal transmission and dengue infection differences between wolbachia strains in aedes aegypti. MBio 12(6):00250–21. 10.1128/mBio.00250-21 PMC8576525 34749528

[CR35] Nazni WA, Hoffmann AA, NoorAfizah A, Cheong YL, Mancini MV, Golding N, Kamarul GMR, Arif MAK, Thohir H, NurSyamimi H, ZatilAqmar MZ, NurRuqqayah M, NorSyazwani A, Faiz A, Irfan F-RMN, Rubaaini S, Nuradila N, Nizam NMN, Irwan SM, Endersby-Harshman NM, White VL, Ant TH, Herd CS, Hasnor AH, AbuBakar R, Hapsah DM, Khadijah K, Kamilan D, Lee SC, Paid YM, Fadzilah K, Topek O, Gill BS, Lee HL, Sinkins SP (2019) Establishment of wolbachia strain walbb in Malaysian populations of aedes aegypti for dengue control. Curr Biol 29(24):4241–42485. 10.1016/j.cub.2019.11.007 PMC6926472 31761702

[CR36] Ogunlade ST, Adekunle AI, Meehan MT, Rojas DP, McBryde ES (2020) Modeling the potential of w au-wolbachia strain invasion in mosquitoes to control aedes-borne arboviral infections. Sci Rep 10(1):16812 10.1038/s41598-020-73819-1 PMC7544821 33033285

[CR37] Onen H, Luzala MM, Kigozi S, Sikumbili RM, Muanga C-JK, Zola EN, Wendji SN, Buya AB, Balciunaitiene A, Viškelis J et al (2023) Mosquito-borne diseases and their control strategies: an overview focused on green synthesized plant-based metallic nanoparticles. Insects 14(3):221 10.3390/insects14030221 PMC10059804 36975906

[CR38] Orozco-Gonzales JL, Santos Benedito A, Cardona-Salgado D, Ferreira CP, Oliveira Florentino H, Sepulveda-Salcedo LS, Vasilieva O (2024) Comparing the long-term persistence of different Wolbachia strains after the release of bacteria-carrying mosquitoes. Math Biosci 372:109190. 10.1016/j.mbs.2024.109190 38631561

[CR39] Parra JR, Coelho A Jr (2022) Insect rearing techniques for biological control programs, a component of sustainable agriculture in Brazil. Insects 13(1):105. 10.3390/insects13010105 PMC8778874 35055948

[CR40] Pavan MG, Gnonhoue FJ, Corrêa-Antônio J, Padilha KP, Garcia GA, Oliveira F, Brito LP, Dias L, Martins AJ, Corbel V, Lima JBP, Wallau GL, Hoffmann A, Cruz OG, Villela DAM, Maciel-de-Freitas R (2025) The long-term persistence of the wmel strain in Rio de Janeiro is threatened by poor integrated vector management and bacterium fitness cost on aedes aegypti. PLoS Negl Trop Dis 19(7):0013372. 10.1371/journal.pntd.0013372 PMC12310003 40700442

[CR41] Pei Y, Chen M, Liang X, Li C, Zhu M (2018) Optimizing pulse timings and amounts of biological interventions for a pest regulation model. Nonlinear Anal Hybrid Syst 27:353–365. 10.1016/j.nahs.2017.10.003

[CR42] Pinto SB, Riback TI, Sylvestre G, Costa G, Peixoto J, Dias FB, Tanamas SK, Simmons CP, Dufault SM, Ryan PA et al (2021) Effectiveness of wolbachia-infected mosquito deployments in reducing the incidence of dengue and other aedes-borne diseases in niterói, brazil: A quasi-experimental study. PLoS Negl Trop Dis 15(7):0009556. 10.1371/journal.pntd.0009556 PMC8297942 34252106

[CR43] Ritchie SA, Townsend M, Paton CJ, Callahan AG, Hoffmann AA (2015) Application of w melpop wolbachia strain to crash local populations of aedes aegypti. PLoS Negl Trop Dis 9(7):0003930. 10.1371/journal.pntd.0003930 PMC4512704 26204449

[CR44] Ross PA (2021) Designing effective Wolbachia release programs for mosquito and arbovirus control. Acta Trop 222:106045 10.1016/j.actatropica.2021.106045 34273308

[CR45] Ross PA, Wiwatanaratanabutr I, Axford JK, White VL, Endersby-Harshman NM, Hoffmann AA (2017) Wolbachia infections in aedes aegypti differ markedly in their response to cyclical heat stress. PLoS Pathog 13(1):1006006. 10.1371/journal.ppat.1006006 PMC5215852 28056065

[CR46] Ross PA, Ritchie SA, Axford JK, Hoffmann AA (2019) Loss of cytoplasmic incompatibility in wolbachia-infected aedes aegypti under field conditions. PLoS Negl Trop Dis 13(4):0007357. 10.1371/journal.pntd.0007357 PMC6493766 31002720

[CR47] Ross PA, Axford JK, Yang Q, Staunton KM, Ritchie SA, Richardson KM, Hoffmann AA (2020) Heatwaves cause fluctuations in w mel wolbachia densities and frequencies in aedes aegypti. PLoS Negl Trop Dis 14(1):0007958. 10.1371/journal.pntd.0007958 PMC6977724 31971938

[CR48] Ryan PA, Turley AP, Hoffmann AA, Massaro J, Kearney S, Endersby NM, White VL, Iturbe-Ormaetxe I, Hurst GDD, Ross PA, O’Neill SL (2020) Establishment of wmel wolbachia in aedes aegypti mosquitoes and reduction of local dengue transmission in cairns and surrounding locations in northern Queensland. Australia Gates Open Res 3:1547. 10.12688/gatesopenres.13061.2 PMC6801363 31667465

[CR49] Santos Benedito A, Ferreira CP, Adimy M (2020) Modeling the dynamics of Wolbachia-infected and uninfected aedes aegypti populations by delay differential equations. Math Model Nat Phenomena 15:76

[CR50] Santos Benedito A, Ferreira CP, Oliveira Florentino H (2023) Establishing the coexistence of Wolbachia-carrying and wild aedes aegypti populations by feedback linearization. Appl Math 17(3):521–533. 10.18576/AMIS/170320

[CR51] Steven W, Ombeni K, Kweka EJ, Kimirei M, Cleopa C (2021) Predator preferences: a key to effective biological control design. J Health Biol Sci 9(1):1–4

[CR52] Sukupayo PR, Poudel RC, Ghimire TR (2024) Nature’s solution to aedes vectors: Toxorhynchites as a biocontrol agent. J Trop Med 2024(1):3529261. 10.1155/2024/3529261 PMC11213640 38948015

[CR53] Ufuktepe U (2022) Discrete Wolbachia diffusion in mosquito populations with Allee effects. Eur J Pure Appl Math 15(4):1613–1622. 10.29020/nybg.ejpam.v15i4.4524

[CR54] Ulrich JN, Beier JC, Devine GJ, Hugo LE (2016) Heat sensitivity of w mel wolbachia during aedes aegypti development. PLoS Negl Trop Dis 10(7):0004873. 10.1371/journal.pntd.0004873 PMC4961373 27459519

[CR55] Velez ID, Uribe A, Barajas J, Uribe S, Ángel S, Suaza-Vasco JD, Duran Ahumada JS, Mejia Torres MC, Arbeláez MP, Santacruz-Sanmartin E, Duque L, Martínez L, Posada T, Patiño AC, Gonzalez SM, Velez AL, Ramírez J, Salazar M, Gómez S, Osorio JE, Iturbe-Ormaetxe I, Dong Y, Muzzi FC, Rances E, Johnson PH, Smithyman R, Col B, Green BR, Frossard T, Brown-Kenyon J, Joubert DA, Grisales N, Ritchie SA, Denton JA, Gilles JRL, Anders KL, Kutcher SC, Ryan PA, O’Neill SL (2023) Large-scale releases and establishment of wmel wolbachia in aedes aegypti mosquitoes throughout the cities of bello, medellín and itagüi, colombia. PLoS Negl Trop Dis 17(11):0011642. 10.1371/journal.pntd.0011642 PMC10688688 38032856

[CR56] Wang X, Tao Y, Song X (2011) Analysis of pest-epidemic model by releasing diseased pest with impulsive transmission. Nonlinear Dyn 65(1):175–185. 10.1007/s11071-010-9882-4

[CR57] Xue L, Fang X, Hyman JM (2018) Comparing the effectiveness of different strains of wolbachia for controlling chikungunya, dengue fever, and zika. PLoS Negl Trop Dis 12(7):0006666. 10.1371/journal.pntd.0006666 PMC6085076 30059498

[CR58] Yu J, Zheng B (2019) Modeling Wolbachia infection in mosquito population via discrete dynamical models. J Differ Equ Appl 25(11):1549–1567

[CR59] Zara ALA, Dos Santos SM, Fernandes-Oliveira ES, Carvalho RG, Coelho GE (2016) Estratégias de controle do aedes aegypti: uma revisão. Epidemiol Serv Saúde 25(2):391–404 10.5123/S1679-49742016000200017 27869956

[CR60] Zardini A, Menegale F, Gobbi A, Manica M, Guzzetta G, d’Andrea V, Marziano V, Trentini F, Montarsi F, Caputo B et al (2024) Estimating the potential risk of transmission of arboviruses in the Americas and Europe: a modelling study. Lancet Planet Health 8(1):30–40. 10.1016/S2542-5196(23)00252-8 38199719

[CR61] Zheng X, Zhang D, Li Y, Yang C, Wu Y, Liang X, Liang Y, Pan X, Hu L, Sun Q et al (2019) Incompatible and sterile insect techniques combined eliminate mosquitoes. Nature 572(7767):56–61. 10.1038/s41586-019-1407-9 31316207

